# S-nitrosylation of UCHL1 induces its structural instability and promotes α-synuclein aggregation

**DOI:** 10.1038/srep44558

**Published:** 2017-03-16

**Authors:** Roshan Kumar, Deepak K. Jangir, Garima Verma, Shashi Shekhar, Pranita Hanpude, Sanjay Kumar, Raniki Kumari, Nirpendra Singh, Neel Sarovar Bhavesh, Nihar Ranjan Jana, Tushar Kanti Maiti

**Affiliations:** 1 Functional Proteomics Laboratory, Regional Centre for Biotechnology (RCB), NCR Biotech Science Cluster, 3rd Milestone Gurgaon-Faridabad Expressway, Faridabad, 121001, India; 2 Manipal University, Manipal, Karnataka, 576104, India; 3 Transcription Regulation Group, International Centre for Genetic Engineering and Biotechnology (ICGEB), Aruna Asaf Ali Marg, New Delhi, 110067, India; 4 Molecular Neuroscience Laboratory, National Brain Research Centre (NBRC), Manesar, Gurgaon, 122051, India; 5 Regional Centre for Biotechnology (RCB), NCR Biotech Science Cluster, 3rd Milestone Gurgaon-Faridabad Expressway, Faridabad, 121001, India

## Abstract

Ubiquitin C-terminal Hydrolase-1 (UCHL1) is a deubiquitinating enzyme, which plays a key role in Parkinson’s disease (PD). It is one of the most important proteins, which constitute Lewy body in PD patient. However, how this well folded highly soluble protein presents in this proteinaceous aggregate is still unclear. We report here that UCHL1 undergoes S-nitrosylation *in vitro* and rotenone induced PD mouse model. The preferential nitrosylation in the Cys 90, Cys 152 and Cys 220 has been observed which alters the catalytic activity and structural stability. We show here that nitrosylation induces structural instability and produces amorphous aggregate, which provides a nucleation to the native α-synuclein for faster aggregation. Our findings provide a new link between UCHL1-nitrosylation and PD pathology.

Parkinson’s disease (PD), one of the most common neurodegenerative movement disorders, is known to cause abnormal motor neuron functions like rigidity, resting tremor and postural instability. It is characterized by a progressive loss of dopaminergic neurons in midbrain particularly substantia nigra^1^,^2^. Majority of PD cases reported are sporadic. However, almost 10% PD cases are familial in nature^3^. Several genes have been identified to cause a familial PD and among them α-synuclein, PINK1, Parkin, DJ-1 and LRRK2 have been well studied^4^,^5^,^6^. Clinical as well as experimental observations support the hypothesis that increased expression of α-synuclein is important for PD pathogenesis. Increased cytoplasmic expression of α-synuclein in aged human brain is one of the major risk factors for PD development^7^. Genome wide association studies reveal that single nucleotide polymorphisms associated with α-synuclein are linked to increase the risk of PD^8^. α-synuclein is a major component of cytoplasmic inclusions called Lewy body (LB) in sporadic PD patient brain. It indicates that α-synuclein plays a decisive role in the pathogenesis of PD^9^,^10^. However, the mechanism underlying the formation of LB remains poorly understood. Mass spectrometry analysis of LB has identified almost 40 proteins of different families^11^. The question remains unclear how other LB proteins regulate or influence α-synuclein aggregation leading to the loss of neuronal function.

Ubiquitin C-terminal hydrolase-L1 (UCHL1) is a deubiquitinating enzyme which is largely expressed in neuron, comprising almost 1–5% of total brain protein and its absence in mice due to intragenic deletions produces neurodegenerative phenotypes^12^,^13^. It is also one of the major components identified in LB of sporadic PD patient^11^. Immunohistochemistry of midbrain section of patient with sporadic PD has shown that α-synuclein and UCHL1 are double positive in LB in nigral DA neurons. It suggests a physical or functional interaction among themselves^14^. The I93M polymorphism of UCHL1 is linked to an increased risk of PD whereas the S18Y polymorphism in UCHL1 reduces susceptibility to PD and Alzheimer’s disease (AD)^15^. The lower stability and loss of catalytic activity of the I93M mutant may be predisposed to enhance oxidative modification of UCHL1^16^. It has also been demonstrated that S18Y mutation confers antioxidant function of UCHL1 and could be protective in PD^17^. Recent studies have demonstrated that farnesylation of UCHL1 induces its association with neuronal or endoplasmic reticulum membrane and regulates intercellular α-synuclein and neurotoxicity^18^. Chemical inhibition of UCHL1 farnesylation reduces the cellular level of α-synuclein and thus improves neuronal cell viability. Biochemical studies have suggested that chemical modification of UCHL1 caused by 4-hydroxyl-2 nonenal (HNE), a lipid hyper oxidation product or modification of prostaglandin metabolites (Δ12 prostaglandin J2) alters the secondary structure and thereby compromises the enzymatic activity of UCHL1. The cysteine residue in crossover loop (Cys-152) is the major target site for prostaglandin modification which has been demonstrated by mutational and NMR spectroscopy studies^19^,^20^. Recent data have demonstrated that mutation of C152A inhibits cyclopentanone prostaglandins modification and conserves hydrolase activity of UCHL1 after treatment of 15d PGJ2. C152A mutant mice show resistant to 15d PGJ2 toxicity compared to wild type mice^21^. 1,2,3,4-tetrahydroisoquinoline (DHBnTIQ) is an endogenous parkinsonism-inducing dopamine derivative that binds to UCHL1 specifically at Cys152 *in vitro*. This modification increases the amount of UCHL1 in the insoluble fraction of SH-SY5Y cells and inhibited its hydrolase activity to 60%, reducing the level of ubiquitin in the soluble fraction of SH-SY5Y cells^22^. S-mercuration of UCHL1 through Cys152 by methylmercury causes inhibition of its catalytic activity and reduction of monoubiquitin level in SH-SY5Y cells^23^. All these observations indicate that Cys152 has an important role in neuronal survival in stress condition. Despite several biochemical and structural analysis of UCHL1 function the question remains unclear of its involvement in PD pathogenesis.

Studies in human post-mortem brain indicate that reactive oxygen/nitrogen species are important in the pathogenesis of sporadic PD. Increasing evidences have indicated that excessive nitric oxide (NO) production contributes to aging and PD development. The mRNA and protein expression of neuronal nitric oxide synthase (nNOS) are age dependent in brain cortex in rat model^24^. Higher levels of nNOS and inducible nitric oxide synthase (iNOS) have been observed in the substantia nigra of PD patient and in animal model^25^. Recent data have suggested that proteins like Parkin, DJ-1, protein disulphide isomerase (PDI), MAP1B, PTEN, GAPDH and Drp-1 undergo S-nitrosylation which is a post translational modification by NO at the cysteine residue of the target protein and protein nitrosylation plays a decisive role in neuronal function. Parkin is involved in ubiquitination of proteins that are important in survival of dopaminergic neurons. Nitrosylation of parkin enhances its E3 ligase activity and promotes its autoubiquitination and subsequently regulates ubiquitination and clearance of parkin substrate^26^. DJ-1 is known to regulate the activity of PTEN which plays a crucial role in neuronal cell death in response to various insults. PTEN phosphatase activity is regulated by trans-nitrosylation where nitrosylated DJ-1 transfers its NO group to PTEN^27^. S-nitrosylation of PDI inhibits its enzymatic property and activates unfolded protein response and thereby attenuates neuronal cell death triggered by ER stress^28^. During the last few years there is an accumulation of evidences where S-nitrosylation of proteins like cdk5, p53, GAPDH, MMP9 MAP1B, MEF2 and XIAP regulates neurodevelopment and neurodegenerative condition^29^,^30^,^31^,^32^,^33^,^34^,^35^.

In the present study, we report that UCHL1 is S-nitrosylated in *in vitro* condition on treatment of S-nitrosogluthione (GSNO). Rotenone treated SH-SY5Y cells enhance NO production thereby promoting S-nitrosylation of UCHL1. We have shown that S-nitrosylation of UCHL1 disrupts its deubiquitinase activity and structural fold which eventually produces amorphous protein aggregates *in vitro*. We have also demonstrated that UCHL1 S-nitrosylation provides seeding for faster aggregation of α-synuclein. Finally, the *in vitro* nitrosylation of UCHL1 was corroborated with rotenone induced mouse model of PD. Our study collectively reveals a new mechanism where we have provided the missing link in UCHL1, Lewy body composition and α-synucleinopathy.

## Results

### S-nitrosylation of UCHL1 in SH-SY5Y cells and *in vitro*

**Figure 1:**
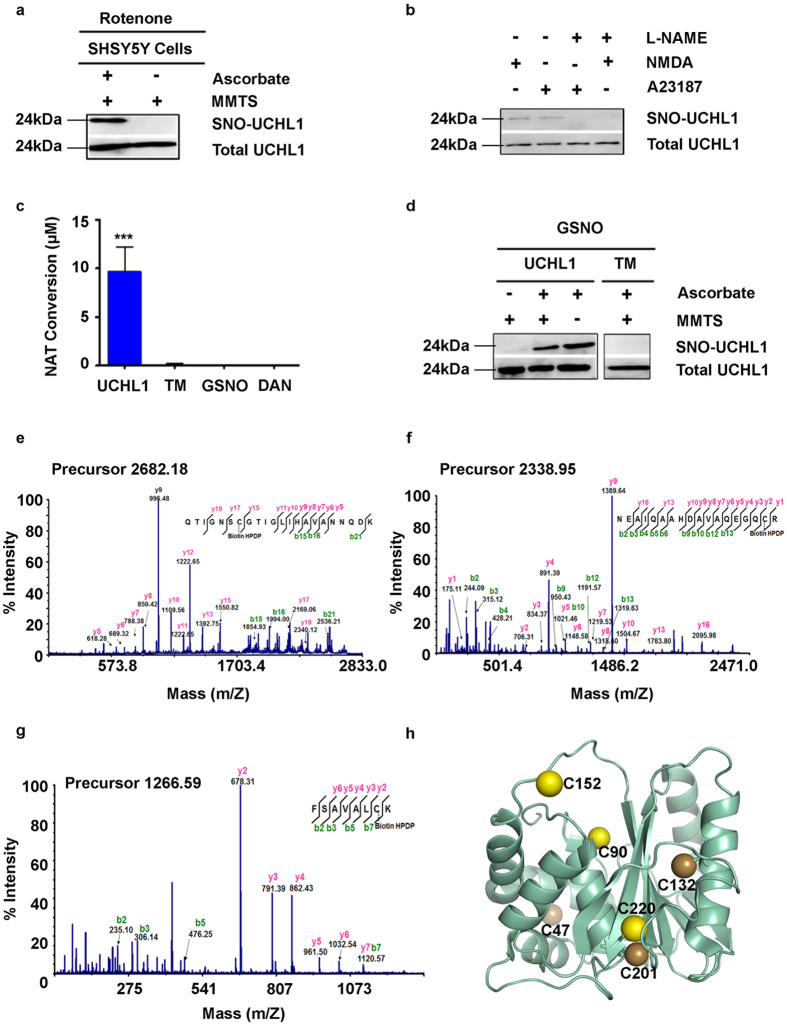
S-nitrosylation of UCHL1 in neuroblastoma SH-SY5Y cells and in recombinant UCHL1. **(a)** Biotin switch assay was performed after 16 h exposure of rotenone (1 μM) to SH-SY5Y cells. Lower panel represents loading control. For specificity of this assay sodium ascorbate (20 mM) was omitted in right lane. MMTS was used for effective blocking of free thiols to minimize background biotinylation and false signal (**b)** nNOS was activated in SH-SY5Y cells by NMDA (50 μM) and calcium ionophore A23187 (5 μM) separately and in presence and absence of NOS inhibitor L-NAME. **(c)** Nitrosylation quantification was done by incubating UCHL1 (10 μM) with GSNO (10 molar excess) for 15 min at 37 °C. S-nitrosylated UCHL1 generated was analyzed by release of NO, causing the conversion of NO to NAT. Values are mean ± s.e.m., n ≥ 3; ***P < 0.0001 by one way analysis of variance with Bonferroni post hoc test. (**d**) Nitrosylation in recombinant UCHL1. Biotin switch assay was performed after incubating 2 mg/ml UCHL1 with 10 molar excess GSNO. Lower panel represents loading control. UCHL1 protein is detected by anti-UCHL1 antibody after streptavidin pull down. **(e–g)** Mass spectrometry (MALDI MS) analysis identifies C90, C152 and C220 as nitrosylation site. MS/MS spectra of trypsin digested peptide fragments **(e)** QTIGNSCGTIGLIHAVANNQDK **(f)** NEAIQAAHDAVAQEGQCR and **(g)** FSAVALCK were shown to be modified with HPDP Biotin. **(h)** Crystal structure of UCHL1 (PDB 2ETL), nitrosylated cysteine shown as yellow spheres and unmodified cysteine as brown spheres. Uncropped western blots were presented in Supplementary Fig. S9.

To investigate the effect of nitrosative stress on UCHL1 function and its impact on cellular regulation, we exposed SH-SY5Y cells with 1 μM of rotenone, a herbicide that disrupts mitochondrial complex I function and generates ROS/RNS species^36^. It has been shown earlier that rotenone treatment to SH-SY5Y cells induces cellular NO production which subsequently leads to the S-nitrosylation of different proteins^37^. We measured the NO content within the cells by using 4-Amino-5-Methylamino-2′,7′-Difluorofluorescein Diacetate (DAF-FM DA), a reagent that reacts with NO to form a fluorescent benzotriazole that could be detected using fluorescence activated cell sorting (FACS). Rotenone treated cells showed almost 10 fold enhanced NO production compared to untreated cells (Supplementary Fig. S1). Using the biotin switch technique (BST) followed by LC-MS/MS analysis in rotenone treated cells revealed that UCHL1 was one of the proteins targeted for nitrosylation (Supplementary Table S1). S-nitrosylation of UCHL1 in rotenone-induced condition was further validated by western blot analysis. The rotenone treated SH-SY5Y cell showed UCHL1 nitrosylation whereas the control cell did not show any nitrosylation. This indicates that the S-nitrosylation of UCHL1 occurred in a nitrosative stress condition only (Fig. 1a).

Excitotoxic damage is a common pathway that is observed in most neurodegenerative disorders and is largely caused by over stimulation of N-methyl -D-Aspartate (NMDA) receptor^38^. Excessive influx of Ca^2+^ ion through the receptor associated ion channel triggers a detrimental enzymatic reaction and generates harmful reactive oxygen and nitrogen species^39^. We treated SH-SY5Y cells with NMDA, an agonist of NMDA receptor, calcium ionophore (A23187) which induces nNOS and also with L-NAME which is an inhibitor of nitric oxide synthase (NOS)^28^,^40^,^41^. UCHL1 nitrosylation was observed in both calcium ionophore and NMDA treated condition. However, this nitrosylation was quenched by addition of L-NAME (Fig. 1b). To identify whether UCHL1 was S-nitrosylated *in vitro* the purified recombinant UCHL1 (2 mg/ml) was treated with GSNO (10 molar excess). We have quantified the nitrosylation of UCHL1 wild type by DAN assay. DAN assay showed almost 10 folds protein S-nitrosylation upon GSNO treatment of UCHL1 wild type (Fig. 1c). The western blot also confirms recombinant UCHL1 nitrosylation upon treatment with GSNO (Fig. 1d). Nitrosylated site identification was done using biotin switch assay coupled with mass spectrometry. Trypsin digested biotinylated peptides were purified by streptavidin agarose resin. The eluted peptides were analyzed by LC-MS/MS analysis. MS/MS analysis confirmed that Cys 90, 152 and 220 of UCHL1 were modified by biotin (Fig. 1e–g) on GSNO treatment. To validate this data, a triple alanine mutant (TM) of these cysteine residues was made. The triple alanine mutant protein (C90A, C152A and C220A) was purified and *in vitro* nitrosylation experiment was performed. TM does not show any signal in DAN assay and in western blot. S-Methyl methanethiosulfonate (MMTS) is an alkylating agent and blocks free thiol group. Effective blocking of free thiols is necessary to minimize background biotinylation and maximize assay sensitivity^42^. Collectively, above findings indicated that increase of cellular nitric oxide promoted UCHL1 nitrosylation of three critical cysteine residues (Fig. 1h).

### S-nitrosylation lowers the enzymatic activity and ubiquitin binding of UCHL1

**Figure 2:**
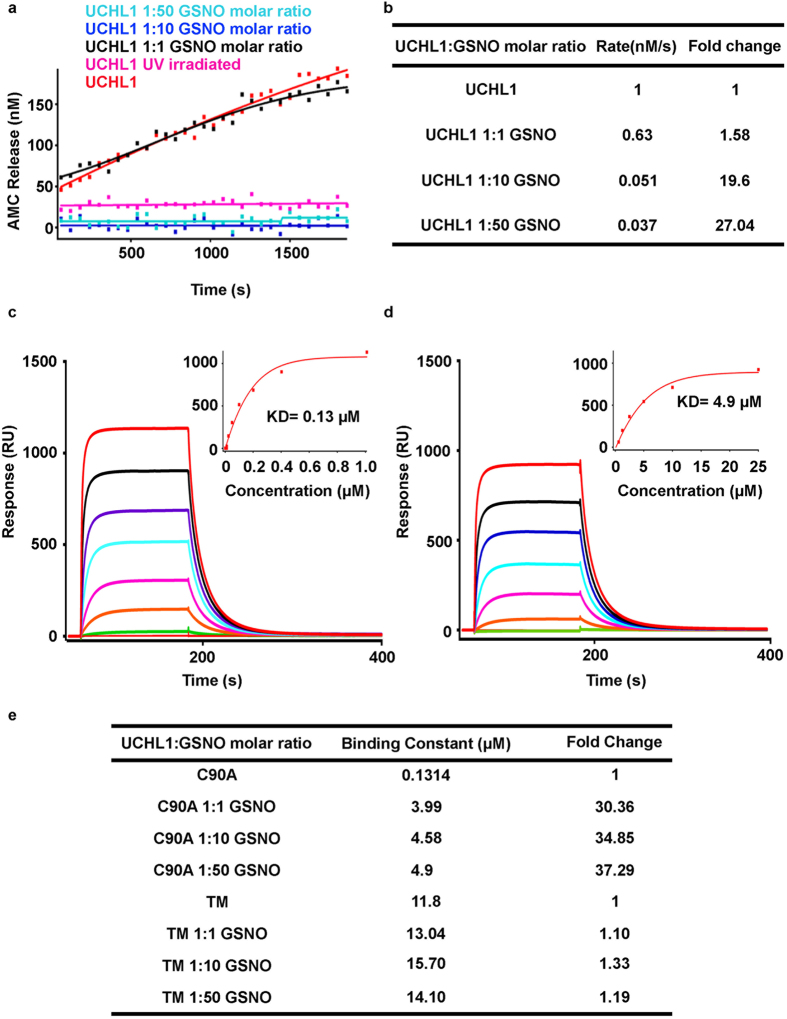
S-nitrosylation of UCHL1 down regulates its catalytic activity and compromises ubiquitin binding. **(a)** Enzymatic activity of the nitrosylated UCHL1. UCHL1 and nitrosylated UCHL1 (2.5 nM) were incubated with Ub-AMC (450 nM). UV irradiation was used to check reversibility of UCHL1 activity after nitrosative modification. Hydrolysis was measured as an increase in fluorescence, indicating release of AMC, over time. **(b)** Values showing the rate of reaction catalyzed by control and nitrosylated UCHL1. **(c,d)** Sensograms correspond to the binding of UCHL1 (analyte) to an immobilized ubiquitin on CM5 biosensor chips (~2200 RU). **(c)** UCHL1 C90A and **(d)** nitrosylated UCHL1 C90A ranging from 0.078 μM to 50 μM were used. Inset represents steady state affinity curve and binding constant (KD) of UCHL1 C90A and nitrosylated UCHL1 C90A which were calculated to be 0.13 μM and 4.90 μM respectively using BIA evaluation software. Sensogram shown in different colors represent different concentrations (Red 50 μM, black 25 μM, purple 12.5 μM, cyan 6.25 μM, pink 3.12 μM, orange 1.56 μM and green 0.78 μM). (**e**) Values showing binding constant (μM) of nitrosylated and control UCHL1 C90A and TM.

To test whether the ubiquitin hydrolase activity of UCHL1 was impaired on S-nitrosylation, an *in vitro* hydrolase activity assay was performed using Ub-AMC as a model substrate^43^. UCHL1 was incubated with varying concentration of GSNO at 37 °C for 30 min and then Ub-AMC substrate was added to each of the reaction. The fluorescence intensity of AMC was monitored which was released from Ub-AMC due to hydrolysis. The activity of UCHL1 was almost abolished on treatment of 10 and 50 molar excess of GSNO (Fig. 2a). There was not much change in rate of hydrolysis in 1eqv GSNO treated condition. However, significant change in rate was observed in 10 and 50 molar excess conditions (Fig. 2b). We also checked the reversibility of UCHL1 activity after nitrosylation by irradiating nitrosylated UCHL1 with UV light for 30 min. Figure 2a demonstrated that the UCHL1 nitrosylation is irreversible in nature. The mass spectrometry data demonstrated that S-nitrosylation of UCHL1 occurred in a dose dependent manner. In one molar excess concentration only Cys 152 residue was nitrosylated, while in 10 molar excess and 50 molar excess conditions Cys 152 and Cys 90 and Cys 220 were nitrosylated (Supplementary Table S2). The loss of catalytic activity of UCHL1 on nitrosylation may be due to the modification of Cys 90 residue or due to compromised ubiquitin binding. To check if the ubiquitin binding was impaired, binding experiments were performed using surface plasmon resonance. UCHL1 C90A and TM were incubated with 50 molar excess of GSNO for 2 h and the treated samples were passed through ubiquitin immobilized CM-5 chip. The varying UCHL1 concentrations ranging from 0–50 μM were used to determine the equilibrium binding constant (Fig. 2c). The binding constant for untreated sample was 0.13 μM, which was very similar to the binding constant observed earlier in isothermal titration calorimetry (ITC). To our surprise equimolar, 10 and 50 molar excess of GSNO treated UCHL1 C90A showed almost 30 fold loss of ubiquitin binding (4.9 μM) compared to wild type (Fig. 2d). The untreated TM also showed a significant loss of binding (almost 20 fold) compared to C90A mutant. But TM mutant did not show any loss of binding on treatment of 50 molar excess of GSNO (Fig. 2e). Our observation unambiguously demonstrated that nitrosylation of these three critical residues prevented Ub-AMC hydrolysis and ubiquitin binding.

### S-nitrosylation of UCHL1 impairs its native structure

**Figure 3:**
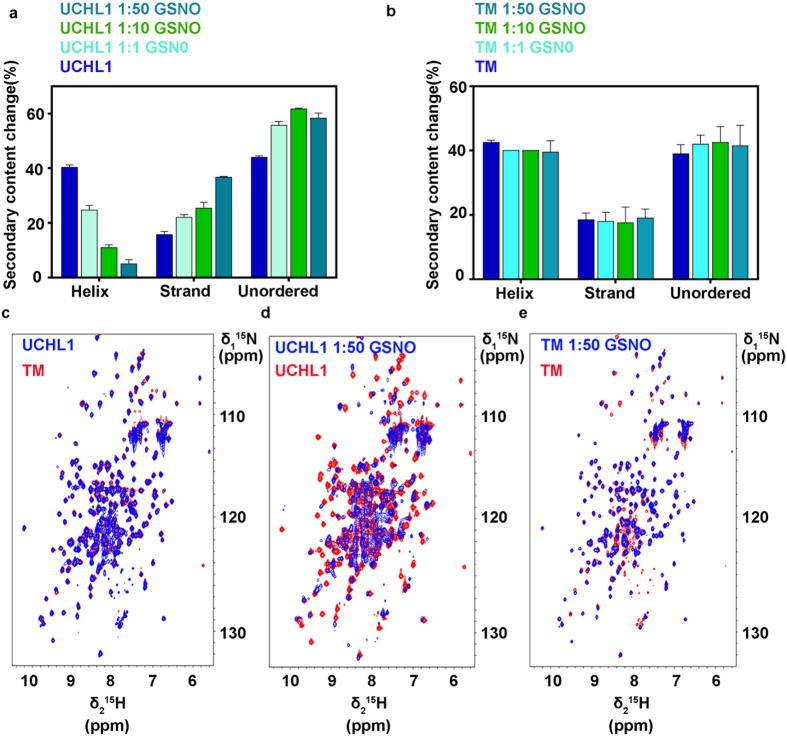
Structural destabilization of nitrosylated UCHL1. Circular dichroism spectroscopy, bars showing secondary structural content of **(a)** UCHL1 and nitrosylated UCHL1 **(b)** TM and nitrosylated TM **(c,d,e)** 2D [^15^N,^1^H] HSQC spectrum of UCHL1 (0.2 mM protein, 100 mM Tris-HCl, pH 7.4, 298 K) exhibiting expected number of resonances with excellent chemical shift dispersion, indicative of a well-folded protein. **(c)** Overlay of 2D [^15^N,^1^H] HSQC spectra of UCHL1 (blue) and TM (red). Substantial line broadening of resonances was observed and poor dispersion of amide proton resonances in a narrow spectral region around 7.5–9 ppm is indicative of partially unfolded/aggregated protein. **(d)** Overlay of 2D [^15^N,^1^H] HSQC spectra of nitrosylated UCHL1 (blue) and UCHL1 (red) **(e)** Overlay of 2D [^15^N,^1^H] HSQC spectra of nitrosylated TM (blue) overlapped with TM as control (red).

To address the structural change, we used circular dichroism spectroscopy to estimate the secondary structural component analysis. UCHL1 wild type and TM were treated with different concentrations of GSNO and the reaction mixture was incubated at 37 °C for 2 h. The CD spectra of all treated condition were measured over the wavelength range from 190 nm to 240 nm (Supplementary Fig. S2a,b). The spectra were analyzed using DICHROWEB online server. The CD spectroscopic data shows that structural changes between WT and TM are within 5% (Helix 40.3% for WT and 42.5% for TM; Strands 15.66% for WT and 18.5% for TM; disordered structure 44% for WT and 39% for TM). There is significant structural change in WT treated with different concentration of GSNO but TM showed resistance to GSNO treatment (Fig. 3a,b). Our observation confirmed that structural destabilization was associated with S-nitrosylation.

To understand the structural change at the molecular level, 2D [^15^N,^1^H] HSQC NMR experiments were carried out with UCHL1 wild type (WT) and TM with 50 molar excess of GSNO and the results were compared with untreated condition. The 2D [^15^N,^1^H] HSQC spectra of WT and TM showed well dispersed resonances with uniform line-widths which is indicative of structurally well folded proteins. Overlay of the 2D [^15^N,^1^H] HSQC spectra of wild type and TM confirmed that the mutations did not change structural conformation (Fig. 3c). On treatment with 50 molar excess of GSNO to wild type caused a dramatic change in 2D [^15^N,^1^H] HSQC spectrum which was an indication of partial unfolding and aggregation of protein (Fig. 3d). In contrast, the overlay 2D [^15^N, ^1^H] HSQC spectrum of TM untreated and 50 molar excess treated conditions did not show any significant change (Fig. 3e). The residue-wise chemical shift perturbation (CSP) is shown in (Supplementary Fig. S3) which indicates there is significant overall structural change in WT compared to TM. The conclusion from CD and NMR analysis proved that nitrosylation induced structural destabilization.

### UCHL1 undergoes amorphous aggregates formation via aberrant nitrosylation

**Figure 4:**
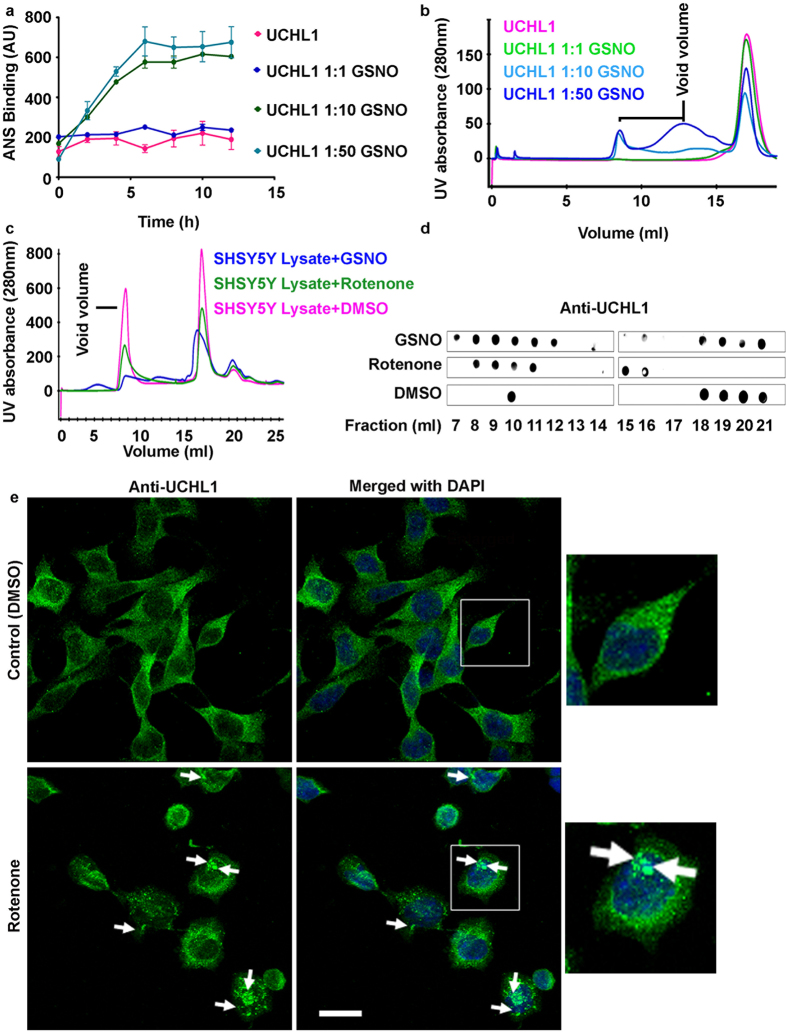
Nitrosative stress induces UCHL1 to undergo amorphous aggregates formation *in vitro* and in cellular condition. **(a)** 8-Anilino-1-naphthalenesulfonic acid (ANS, 20 μM) binding showed hydrophobic surface exposure of UCHL1 with varying concentration of GSNO. **(b)** Size exclusion chromatographic (Superdex 200, 10/30) profile of nitrosylated UCHL1. The void region.is marked with line. **(c)** SH-SY5Y cells treated with GSNO and rotenone (1 μM) for 16 h, lysed and supernatant was loaded to size exclusion chromatographic (Superose 6 10/300 GL) column. **(d)** Different fractions obtained from gel filtration chromatography were analyzed using dot blot analysis against anti-UCHL1 antibody. **(e)** Confocal images showing aggregates (marked with arrow) in rotenone treated neuroblastoma SH-SY5Y cells but not in control cells. Scale bar represents 20 μm.

To verify the structural destabilization of UCHL1, l-Anilinonaphthalene-8-sulphonate (ANS) binding experiment was performed. ANS measures the surface hydrophobicity in the proteins. The quantum yield of ANS increased significantly after binding to the hydrophobic portions of proteins and suggested the use of ANS as a hydrophobic probe for the study of conformational changes in a given protein. The fluorescence dye ANS is a valuable probe for the detection and analysis of conformational changes in proteins^44^. UCHL1 was treated with equimolar, 10 and 50 molar excess of GSNO and the hydrophobic surface exposure was monitored. Untreated UCHL1 and equimolar GSNO treated UCHL1 did not show any change of ANS binding whereas 10 and 50 molar excess of GSNO treated UCHL1 showed a very high ANS binding (Fig. 4a), indicating a hydrophobic collapse due to nitrosylation.

To confirm if structural destabilization induced UCHL1 aggregation due to nitrosylation, 50 molar excess of GSNO treated sample was subjected to size exclusion chromatography. Untreated UCHL1 behaved like a monomeric protein which appeared in molecular weight range of 26 kDa whereas treated sample exhibited a new peak near void volume in elution chromatogram which indicates the presence of higher molecular weight aggregated species (Fig. 4b). In order to evaluate whether our *in vitro* observations were compatible to cellular UCHL1 aggregation, size exclusion chromatography of SH-SY5Y cells were performed with two different conditions. Cells were treated with rotenone for 24 h and harvested cells were loaded on to gel filtration column. In another condition, harvested SH-SY5Y cells were treated with 50 molar excess of GSNO for 2 h incubation at 37 °C and treated cells were loaded on to gel filtration chromatography (Fig. 4c). The presence of UCHL1 in each chromatography fractions was tested using anti UCHL1 antibody in dot blot experiment. UCHL1 in the untreated cells was eluted in region of 26 kDa whereas UCHL1 was eluted mainly in void volume in both rotenone and GSNO treated condition (Fig. 4d). This observation indicated that rotenone or GSNO treatment induced cellular aggregation.

We have validated cellular aggregation of UCHL1 on rotenone treatment using confocal microscopy. SH-SY5Y cells were grown on a cover slip and cells were treated with rotenone for 24 h. UCHL1 in fixed cells was visualized by immunostaining using anti UCHL1 antibody. Rotenone treated cells showed increased cytoplasmic aggregation compared to untreated cells (40% in treated cells and 20% in normal cells) (Fig. 4e, Supplementary Fig. S4). Our *in vitro* and cellular results have unambiguously established that UCHL1 undergoes aggregation upon nitrosylation.

### Amorphous UCHL1 aggregates promoted α-synuclein fibrillogenesis

As UCHL1 is one of the important components in LB it is pertinent to study if nitrosylation induces fibrillization of UCHL1 and whether nitrosylated UCHL1 influences/induces aggregation of α-synuclein. First, we tested the morphology of nitrosylation induced UCHL1 aggregates using atomic force microscopy (AFM). UCHL1 was treated with 50 molar excess of GSNO at 37 °C for 2 h in dark and the solution was centrifuged at 10,000 × g for 1 h. The protein pellet was washed with MiliQ water and the sample was placed on freshly cleaved mica. AFM image was taken in a noncontact mode. AFM image of nitrosylated UCHL1 showed amorphous aggregates whereas untreated UCHL1 did not show any aggregation (Supplementary Fig. S5).

**Figure 5:**
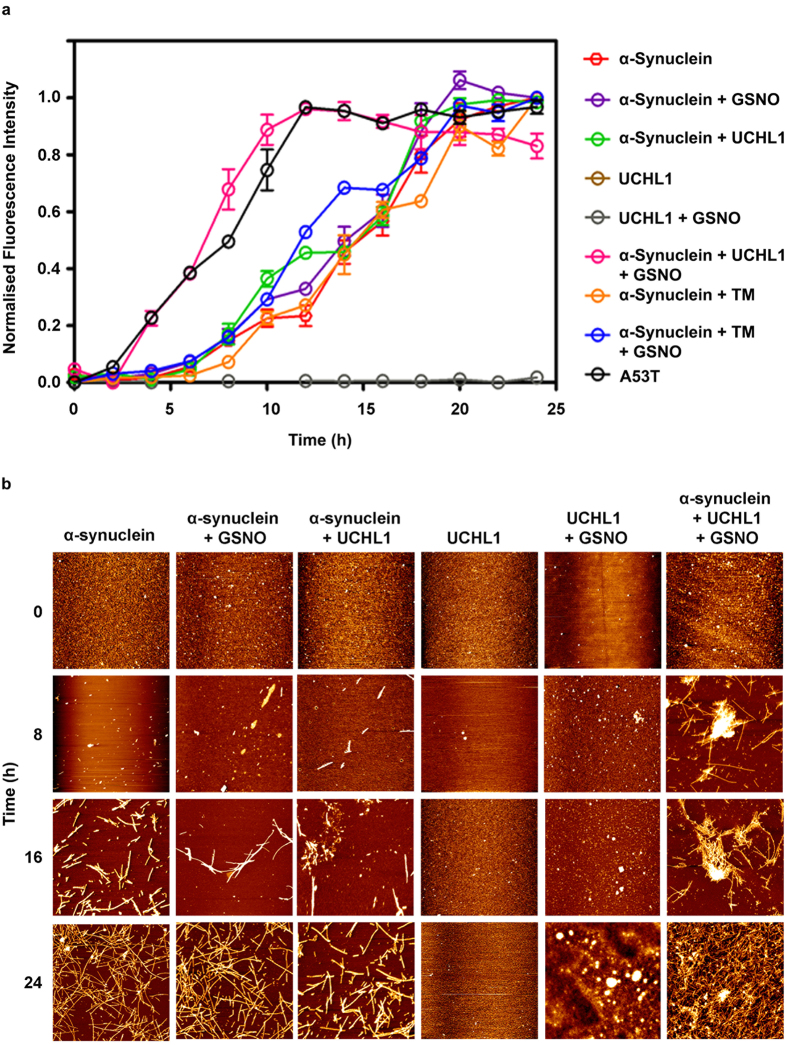
Cross seeding of nitrosylated UCHL1 to α-synuclein. Nitrosylated UCHL1 promoted surface assisted nucleation of α-synuclein and accelerated its fibrillation. (**a**) Time-course quantification of amyloid fibril formation by Thioflavin-T (ThT) binding assay. UCHL1 (200 μM) with 10 molar excess of GSNO accelerated fibrillation of α-synuclein by decreasing the lag phase. TM in nitrosative stress condition behaved like UCHL1 wild type and showed no effect on lag phase, confirming that only nitrosylated UCHL1 promotes α-synuclein fibrillation. (**b**) Samples at 0 h, 8 h, 16 h, and 24 h at 37 °C were aliquoted and processed for atomic force microscopy. The nitrosylated UCHL1 showed fibrils at 8 h but monomeric or oligomeric structures were observed in other samples at same time point.

To probe the influence of nitrosylated UCHL1 on aggregation propensity of α-synuclein we first investigated the interaction between nitrosylated UCHL1 and α-synuclein using Thioflavin T assay (ThT). The aggregation process was monitored over the period of 24 h. Samples at different time points were taken and ThT binding assay was monitored by measuring the ThT fluorescence change. UCHL1 and nitrosylated UCHL1 did not show any change in ThT fluorescence. The α-synuclein mixed with UCHL1 and α-synuclein alone showed ThT fluorescence change after 8 h and reached saturation at 24 h. However, α-synuclein mixed with UCHL1 and 10 molar excess of GSNO showed rapid change of ThT fluorescence (within 2 h) and reached saturation within 10 h which was similar to A53T mutant of α-synuclein. When UCHL1 TM was incubated with α-synuclein and 10 molar excess of GSNO, it showed similar kinetics to α-synuclein/UCHL1-α-synuclein without nitrosative stress condition (Fig. 5a). Thus our ThT binding data unequivocally prove that nitrosylated UCHL1 promotes α-synuclein aggregation and it behaves like its PD associated A53T mutant. ThT binding kinetics data was validated using time dependent AFM experiment. AFM image of all samples at 0 h, 8 h, 16 h, and 24 h were analyzed. Interestingly, fibril was observed in nitrosylated UCHL1-α-synuclein sample at 8 h time period which eventually formed condense fibril at 24 h. Both α-synuclein and α-synuclein -UCHL1 samples showed aggregation at 16 h which matured at 24 h (Fig. 5b). TM and GSNO treated TM showed similar behavior like wild type UCHL1 (Supplementary Fig. S6). AFM data nicely corroborated with ThT results.

### S-nitrosylation of UCHL1 induces Parkinson’s disease phenotype in mouse model

**Figure 6:**
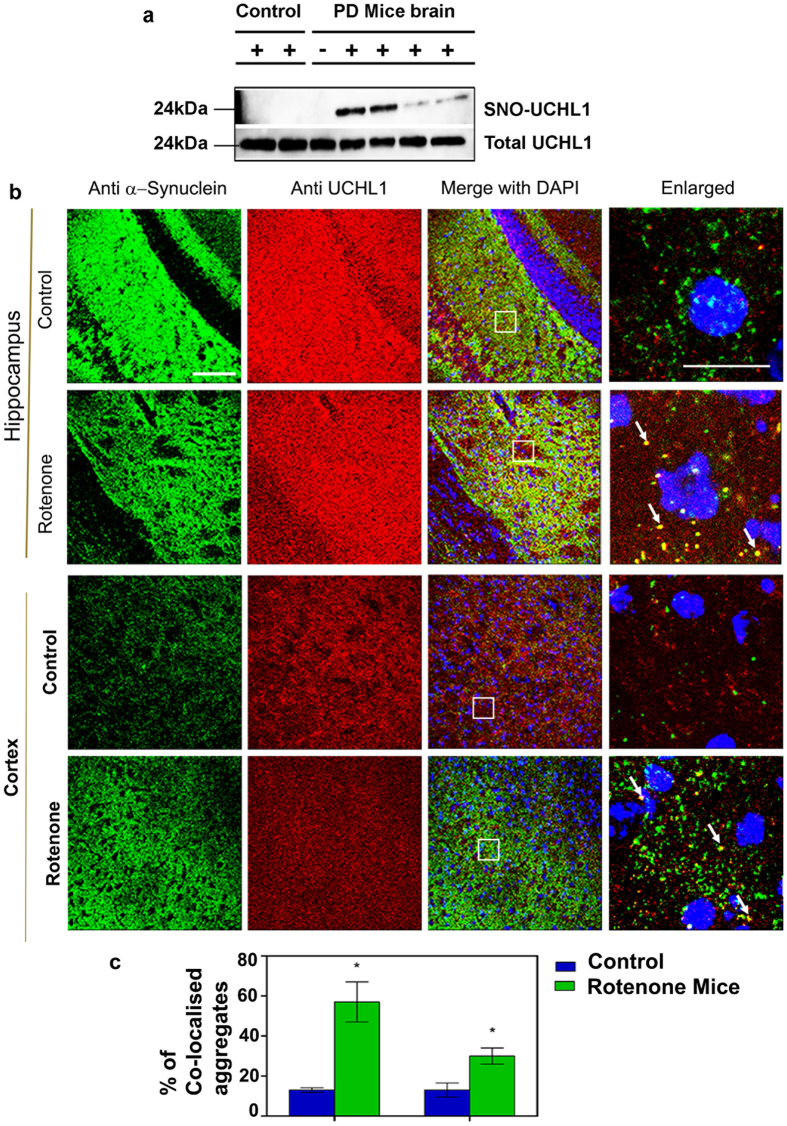
Rotenone induced Parkinson’s mice model showed UCHL1 nitrosylation and aggregation. (**a,b**) C57BL/6 N mice treated orally for 45 days with rotenone (40 mg/kg) body weight. (**a**) Biotin switch assay performed with brain lysate showed UCHL1 nitrosylation in PD mice model brain but not in control brain. Lower panel represents loading control. For specificity of this assay sodium ascorbate (20 mM) was omitted in one of the sample. (**b**) Representative images of UCHL1 and α-synuclein double-immunofluorescence staining of 10-week-old PD and control mice brain in hippocampus and cortex. Alexa Fluor-488-conjugated secondary antibody was used to label α-synuclein (green) and Alexa Fluor 594-conjugated secondary antibody was used to detect UCHL1 (red). Magnified images of α-synuclein and UCHL1 co-localization marked by arrow. Square box represents magnified images. Scale bar: 50 μm (**c**) Approximate number of α-synuclein and UCHL1 co-localized aggregates in hippocampus and cortex of PD and control mice. α-synuclein and UCHL1 co-localized aggregates were counted in 63X oil objective lens. Immunohistochemically stained images (0.2 × 0.2 mm area) were obtained from three different fields in each region. Values were analyzed by two-way ANOVA followed by Benferroni’s post hoc test. *P ≤ 0.01 in comparison with control mice. Uncropped western blots were presented in Supplementary Fig. S9.

Our *in vitro* and cell culture based studies demonstrated that S-nitrosylation of UCHL1 hindered its cellular function through destabilization of native structure and subsequent cellular aggregation. That may be one of the possibilities of being an important component of Lewy bodies in PD patient. To validate S-nitrosylation of UCHL1 in PD condition we employed rotenone induced mouse model. Twelve mice were equally divided into two groups: one was control and other group was rotenone treated. Rota rod test demonstrated that all treated mice showed PD phenotype after 45 days of treatment (Supplementary Fig. S7). Biotin switch assay was performed with mice brain of control and treated samples. We observed that treated mice brain displayed a high level of UCHL1-SNO whereas control did not show any UCHL1-SNO (Fig. 6a). To revalidate S-nitrosylation in mice brain, the biotin-switch mouse brain sample was digested and streptavidin purified peptide sample was analyzed in LC MS/MS. The analyzed mass spectrometry data clearly confirmed S-nitrosylation of UCHL1 *in vivo* (Supplementary Table 1). Next, we attempted to validate our hypothesis if α-synuclein aggregation was induced by nitrosylated UCHL1 *in vivo*. The control and treated mice hippocampus and cortex were immunostained with anti-UCHL1 and anti-α-synuclein antibodies. The rotenone treated mice hippocampus and cortex showed cytoplasmic protein aggregate where both α-synuclein and UCHL1 were present (Fig. 6b,c). It is noteworthy to mention that α-synuclein-UCHL1 aggregation axis may be the potential explanation for development of PD pathology in mice induced by environmental toxins like rotenone.

## Discussion

In the present study we demonstrated that SH-SY5Y cell produces a high level of nitric oxide in the rotenone induced condition which subsequently S-nitrosylates the cysteine residue of UCHL1 and regulates its cellular function. Even in the excitotoxic condition UCHL1 is S-nitrosylated. *In vitro* nitrosylation of UCHL1 followed by mass spectrometry based identification reveals that three critical cysteines, Cys 90, Cys 152 and Cys 220 of UCHL1 were modified by NO. Earlier mass spectrometry based quantitative proteomics also have identified all these cysteine residues modification in different *in vitro* nitrosylating reagents in SH-SY5Y and HEK293 cells^45^,^46^. In the present study we address how UCHL1 nitrosylation impacts on UCHL1 cellular function and pathogenesis of PD. Among these three cysteine residues, Cys 152 which is present in the cross-over loop, plays a major role in the oxidative stress condition. The carbonyl modification by 4-hydroxy 2-nonenal has been observed only in the Cys 152 residue^19^. UCHL1 is also shown to be modified by 15d-PGJ2 (or other cyclopentenone prostaglandins) at specifically Cys 152 residue^20^. This modification does not happen to all cysteine-containing proteins. It indicates that Cys modification is a specific event for this protein’s functional regulation. Structural and biophysical studies of prostaglandin modification of UCHL1 are demonstrated to be completely destabilized from native structure. This is one of the rare examples where one amino acid modification in the loop residues destabilizes the protein structure, which eventually produces protein aggregates^20^. Additionally, a recent knock-In (KI) UCHL1 C152A study in mouse has also demonstrated that this KI mouse is resistant to 15d-PGJ2 neuronal cytotoxicity^21^. The sequence alignment of all four UCH members (UCHL1, UCHL3, UCHL5 and BAP1) reveals that Cys 152 is unique in UCHL1 and this residue is conserved among species (Supplementary Fig. 8a,b). As this protein is highly prevalent in brain, it could be one of the components to neutralize redox stress. In the present study we also have demonstrated that the nitric oxide modification which is likely to happen in nitrosative stress or excitotoxicity condition destabilizes the UCHL1 structure and induces the aggregation of UCHL1. However, how this small NO residue modification has induced the aggregation is still not clear.

Oxidative modifications and down-regulation of UCHL1 is associated with idiopathic PD^47^. The loss of activity of UCHL1 is also correlated to structural destabilization indicated in our SPR binding studies. TM is resistant to nitrosative stress *in vitro* condition. Our solution-state NMR spectroscopy along with CD data has confirmed that the structural loss is due to nitrosylation. The protein aggregation diseases are primarily associated with the formation of beta amyloid aggregation, which causes neuronal toxicity. Our AFM data has demonstrated that nitrosylation induced aggregation of UCHL1 produces amorphous aggregates (Supplementary Fig. S4). Recent data have shown that amorphous protein aggregates can also cause cellular toxicity and induce disease progression. The proteomics analysis of LB from PD patient reveals that UCHL1 co-enriches with α-synuclein, a major constituent of LB and the role of α-synuclein in PD progression needs no clarification^11^. α-Synuclein mutations enhance membrane binding and aggregation rate which eventually produce amyloid aggregate. Lewy body component DJ1, which is a redox dependent molecular chaperone, prevents α-synuclein aggregation and protects neurons from α-synuclein related toxicity^48^. S-nitrosylation of DJ1 in its critical residues (Cys46 and Cys53) in nitrosative stress condition inhibits its antioxidant function in dopaminergic neurons rendering them for highly susceptible in sporadic PD. Hsp 70 also works as a disaggregase and disassembles α-synuclein aggregates to nontoxic component. There are only a few reports where enhanced aggregation of α-synuclein has been reported by the native proteins. The Peptidyl-Prolyl Isomerases (PPIases) namely FKBP12, FKBP52 and FKBP65 accelerate the aggregation of α-synuclein *in vitro* and in a neuronal cell culture model for synucleinopathy^49^. Our work in *in vitro* aggregation assay (ThT binding and AFM) demonstrates that S-nitrosylation of UCHL1 induces the unfolding of structure and slowly transforms to amorphous aggregates which eventually provide a nucleation for α-synuclein to aggregate in a faster rate. Our findings have revealed a new mechanism why UCHL1 is one of the components present in Lewy bodies along with α-synuclein. The hippocampus and cortex region of rotenone induced PD mice model have revealed that both UCHL1 and α-synuclein co-localize as protein aggregates. However, a detailed molecular as well as biochemical and biophysical work will be necessary to elucidate the complete mechanism.

**Figure 7:**
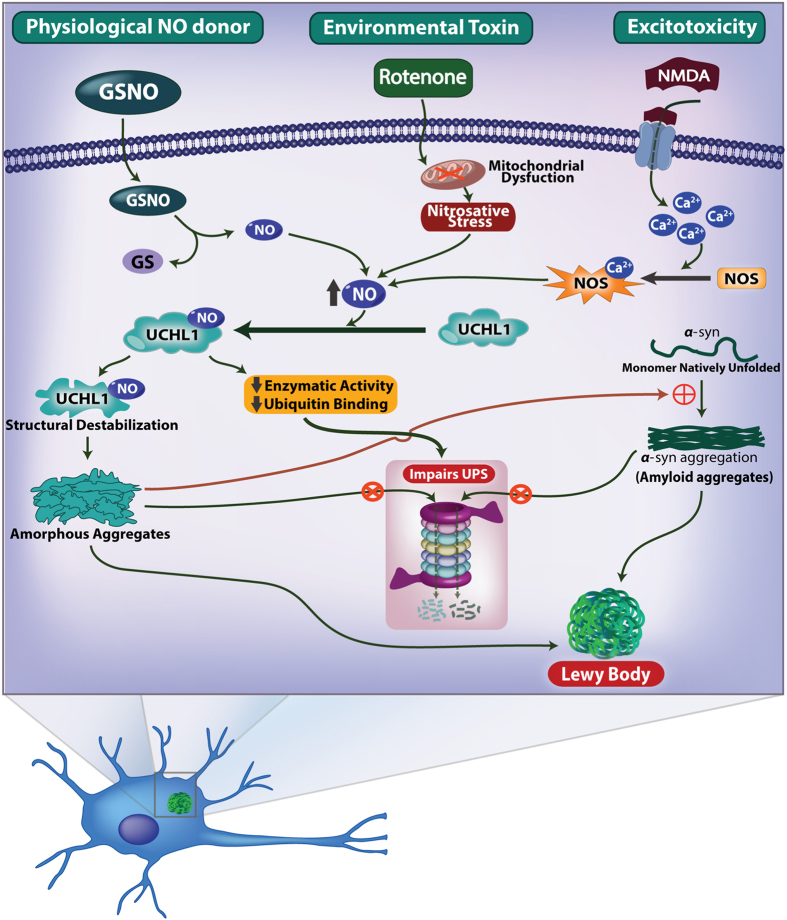
Schematic model. The schematic describes the proposed model for involvement of UCHL1 in ubiquitin proteasome system impairment and acceleration of α-synuclein fibrillation.

In summary, in this study we have demonstrated that nitric oxide which is generated either in nitrosative stress or excitotoxicity conditions or due to mitochondrial dysfunction, S-nitrosylates UCHL1 and hinders its deubiquitinating activity and promotes α-synuclein aggregation promoting Parkinson’s disease (Fig. 7). Thus the elucidation of S-nitrosylation of UCHL1 and its impact on α-synuclein aggregation may provide a new therapeutic target for PD in general.

## Materials and Methods

### Cloning, expression and purification of UCHL1

Flag-HA tagged full length UCHL1 was obtained from Addgene, USA. Full length UCHL1 was sub-cloned into pGEX-6P-1 vector using standard cloning procedure. Mutants of UCHL1 were generated by site directed mutagenesis using Quick-change site directed mutagenesis kit (Stratagene, USA). Wild type UCHL1 and all mutants were expressed in *E. coli* Rosetta 2 cells (Novagen, USA) and proteins were purified according to the standard GST purification protocol. Briefly, Rosetta 2 cells containing pGEX-6P-1 UCHL1 plasmid were grown to 0.6 O.D. in LB medium and then induced with 1 mM of IPTG at 18 °C for 18 h. Cells were then harvested and pellet was resuspended in 1x PBS and 400 mM KCl buffer, pH 7.4 and homogenized. The homogenized cells were centrifuged at 16,000 × g for 45 min and the clear supernatant was loaded onto a GST affinity column (GE Healthcare, USA). Protein was eluted in elution buffer (50 mM Tris-HCl, pH 8.0, 500 mM NaCl and 20 mM reduced glutathione) and dialyzed extensively with 1x PBS and 400 mM KCl buffer, pH 7.4. The GST tag was removed using Pre-scission protease (GE Healthcare, USA). Finally the protein was purified in superdex-75 16/600 gel filtration column. The purity of protein was assessed using SDS-PAGE.

### Rotenone induced Parkinson mice model

All animal experiments were conducted in accordance to the guidelines of animal care facility of National Brain Research Centre and were approved by the animal ethics committee at NBRC. Eight weeks old C57BL/6 N mice (20–28 g) were acclimated to 12 h light/dark cycle and maintained at 23 °C. Mice were housed in standard laboratory cages and had free access to food and water throughout the study. Rotenone (40 mg/ml) was suspended in 0.5% carboxymethyl cellulose sodium salt and administered orally at a volume of 5 ml/kg body weight, once daily for 45 days^50^. Mice were either anesthetized and transcardially perfused with PBS containing 4% PFA (w/v) or sacrificed by cervical dislocation and brain parts were stored at −80 °C for further experiments.

### *In vivo* S-nitrosylation assay

S-nitrosylated proteins were analyzed by biotin-switch assay^51^. Briefly, 1 g of brain tissue sample was homogenized in 2 ml of ice-cold HEN buffer (250 mM Hepes, pH 7.7, 1 mM EDTA) containing 0.1 mM neocuproine and halt protease inhibitor (Thermo Fisher Scientific, USA). The protein extract was clarified by centrifugation at 16,000 × g for 20 min at 4 °C. The protein concentration was estimated by BCA method and the protein extract was diluted to final concentration of 1 mg/ml with HEN buffer. The free sulphydryl group in protein was modified by addition of alkylating reagent (250 mM Hepes, pH 7.7, 1 mM EDTA, 0.1 mM neocuproine, 2.5% SDS and 25 mM S-methyl-methanethiosulphonate) and incubated at 50 °C with frequent vortexing. Alkylated protein was precipitated by adding three volume of acetone. The pellet was washed twice with 70% acetone and dissolved in HEN buffer (240 μl, 250 mM) supplemented with 1% SDS, sodium ascorbate (30 μl, 200 mM) and HPDP biotin (30 μl, 2.5 mg/ml). The biotin labeling reaction was quenched after 90 min by precipitating the protein with three volumes of ice cold acetone. Mass spectrometry based nitrosoproteome analysis of mouse brain was carried out by alkylating biotin switch assay (Supplementary methods).

### *In vitro* S-nitrosylation assay

GSNO was chemically synthesized according to published literature^52^. Briefly, reduced glutathione (1.5 g) dissolved in 5.9 ml of 1 M HCl and NaNO_2_ (0.3 g, freshly dissolved in 1 ml water) was mixed with it. The mixture was incubated at room temperature in dark for 30 min and pH of the solution was adjusted to 7.4 using 10 M NaOH. Purified recombinant protein (0.5–2 mg) was incubated with 1, 10 and 50 molar excess of GSNO at 37 °C in dark for 30 min and protein was precipitated by adding three volume of ice cold acetone. The sample was alkylated and biotinylated according to the procedure used for *in vivo* S-nitrosylation assay. These modified protein samples were subjected to MALDI MS/MS nitrosylation site identification and immuno-blot analysis.

### MALDI MS/MS of biotinylated UCHL1

Biotinylated protein pellet was resuspended in 50 mM ammonium bicarbonate buffer pH 8.0 and treated with 1:20 μg trypsin: protein at 37 °C for 18 h. Trypsinized protein was mixed with streptavidin beads and incubated overnight at 4 °C and washed five times with washing buffer (25 mM Hepes, pH 7.5, 600 mM NaCl, 1 mM EDTA and 0.5% Triton X-100) and biotinylated peptides were eluted with elution buffer (100 mM NaCl, 20 mM Hepes, pH 7.7, 1 mM EDTA and 100 mM 2-mercaptoethanol). Eluted peptides were diluted in 500 μl of water and desalted using C18 tip (Pierce, Thermo scientific) following manufacturer’s instructions. Desalted peptides were mixed with CHCA (1:1) MALDI matrix and spotted on a MALDI plate. For MALDI peptide mass fingerprinting (PMF) and MS/MS analysis, samples were processed in ABSciex 5800 MALDI TOF/TOF mass spectrometer in positive ion reflector mode with ion acceleration voltage 25 KV for MS acquisition and 1 KV for MS/MS. The normalized collision energy was set to 35% for precursor ion fragmentation. The MS and MS/MS spectrum were analyzed in MASCOT (version 2.3). The search parameters to detect differential modification were set to 57 amu for carboxyamido methylation, 428 amu for biotinylation and 16 amu for methionine oxidation.

### Immuno-blot assay

The protein pellet after biotin switch assay was resuspended in HENS buffer (250 μl per mg of protein) and mixed with 3 volume of neutralization buffer (25 mM Hepes, pH 7.5, 100 mM NaCl, 1 mM EDTA and 0.5% Triton X-100). Streptavidin agarose beads were added to the protein solution (50 μl of streptavidin agarose beads per mg of protein) and incubated for overnight at 4 °C. Beads were washed five times with washing buffer (25 mM Hepes, pH 7.5, 600 mM NaCl, 1 mM EDTA and 0.5% Triton X-100) and protein was eluted with 20 μl of elution buffer (20 mM Hepes, pH 7.5, 1 mM EDTA, 100 mM NaCl and 2-mercaptoethanol). The eluted protein was mixed with equal volume of 2X SDS-PAGE sample buffer and was loaded onto 15% SDS-PAGE. The protein was transferred to PVDF membrane using Trans-Blot turbo apparatus (Bio-Rad, USA). The PVDF membrane was blocked with 5% skimmed milk for 2 h and further incubated with anti-UCHL1 primary antibody (1:2,000) for overnight at 4 °C. Subsequently, it was probed with anti-mouse secondary antibody (1:10,000) for 45 min. The membrane was developed using Luminata Forte reagent (Millipore, USA) and visualized under chemiluminescence system Image Quant (GE Healthcare, USA).

### 2,3-Diaminonapthalene (DAN) assay

DAN reacts with NO to form napthotriazole (NAT) which can be quantified by fluorescence spectroscopy^53^. *In vitro* nitrosylation of UCHL1 and TM was carried out by incubating equal volume of protein (20 μM) and GSNO solution (1 mM) in 1:50 molar ratio at 37 °C for 30 min. After nitrosylation, excess GSNO was removed by passing the sample through Bio-spin P6 column (Bio-Rad, USA). Nitrosylated protein sample was divided into two equal parts: one part was treated with DAN (150 μM) and other part was treated with DAN (150 μM) and copper acetate solution (150 μM). The reaction mixture was incubated for 30 min in dark and finally the volume was adjusted to 200 μl by adding 0.1 M NaOH. The fluorescence data was collected using Spectramax plate reader M5 (Molecular device, USA) with excitation wavelength at 375 nm and emission at 450 nm. GSNO was used to prepare standard curve for quantification of NO release.

### Enzymatic activity assay

UCHL1 (25 μl, 1 μM) was treated with different molar ratio of GSNO (1:1, 1:10 and 1:50) in 50 mM Tris-HCl, pH 7.4 and 1 mM EDTA for 30 min at 37 °C. Ub-AMC (450 nM, 75 μL) was incubated with control and nitrosylated UCHL1 (2.5 nM) and the rate of Ub-AMC hydrolysis was monitored for 30 min at 30 °C using Spectramax plate reader i3x (Molecular devices, USA). The excitation and emission wavelengths were 360 nm and 485 nm respectively. The amount of AMC release was quantified using 7-aminomethyl coumarin (Sigma, USA) as a standard^43^.

### Ubiquitin binding assay by surface plasmon resonance

UCHL1-ubiquitin binding experiments were performed on a BIAcore T200 instrument (GE Healthcare Life Sciences, USA) at 25 °C. Ubiquitin was immobilized on CM5 chip in a solution of 10 mM sodium acetate, pH 4.5 by amine coupling which gave a response of 2400 response units (RUs). The binding assay was performed in running buffer (50 mM Tris-HCl, 150 mM NaCl, pH 7.4) at a rate of 30 μL/min. UCHL1 C90A (300 μL, 0.1 mM) was treated with varying concentrations of GSNO (300 μL, 0.1 mM, 1 mM and 5 mM) at 37 °C for 30 min. The final protein concentration was 50 μM after GSNO treatment, which was further diluted with 50 mM Tris-HCl, 150 mM NaCl, pH 7.4 to make different concentrations ranging from 0.078 μM to 50 μM. Varying concentrations of UCHL1 C90A and TM were passed through the chip to get the equilibrium steady state binding affinity^54^. The binding affinity (KD) was calculated using steady state affinity binding model, BIAcore T200 evaluation software (v.1.0).

### Solution-state NMR spectroscopy

The NMR sample contained 0.2 mM UCHL1-^15^N-labeled protein in 100 mM Tris-HCl buffer pH 7.4, 100 mM NaCl, 5% D_2_O (*v/v*), 0.02% NaN_3_ (*w/v*). All NMR spectra were measured at 298 K on Bruker *AvanceIII* spectrometers equipped with 5 mm cryogenic triple resonance TCI probes, operating at field strengths of 500 and 700 MHz. The spectrum was acquired with 16 scans per t_1_ increment in ^15^N dimension. Acquisitions times for ^15^N (SW = 1520 Hz) and ^1^H (SW = 7002 Hz) dimension were 105.2 ms (t_1max_) and 146.2 ms (t_2max_) respectively. All spectra were referenced to DSS^55^, processed with Topspin 2.1 (Bruker AG) and data was analyzed using CARA^56^. To identify the possible structural changes on the protein upon addition of GSNO, chemical shift perturbations (CSP) were calculated from the unbound and bound 2D [^15^N,^1^H] HSQC spectra using the following equation^57^;


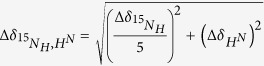


where, Δ*δ*(^1^H^N^) and Δ*δ*(^15^N) are the changes in backbone amide chemical shifts for ^1^H^N^ and ^15^N respectively.

### Circular dichroism spectroscopy

UCHL1 was treated with varying molar ratios of GSNO (1:1, 1:10 and 1:50) and the samples were diluted with 50 mM phosphate buffer, pH 7.2 to reach a final concentration of 10 μM. Circular dichroism spectra were recorded from 190 nm to 260 nm wavelength range using JASCO J815 CD spectrophotometer. The spectra were analyzed by DICHROWEB server for secondary structure content^58^,^59^.

### Size exclusion chromatography and dot blot analysis

Recombinant UCHL1 (200 μl, 250 μM) was treated with different concentrations of GSNO for 2 h and loaded onto a Superdex 200, 10/30 (GE Healthcare Life Sciences, USA) gel filtration column. In case of cellular UCHL1 study, SH-SY5Y cells were treated with GSNO (500 nM) and rotenone (1 μM) for 16 h. Harvested cells were lysed in RIPA buffer (Sigma, USA) containing 1x Halt protease (Thermo Fisher Scientific, USA) and centrifuged at 16,000 × g for 20 min. The supernatant (200 μL, 1.5 mg/mL) was loaded onto a Superose 6 10/300 GL (GE Healthcare, USA) gel filtration column. Each chromatographic fraction was spotted on a nitrocellulose membrane using Bio-Rad dot blot apparatus. The membrane was incubated in 5% skimmed milk for 1 h. After blocking, membrane was incubated with anti-UCHL1 antibody (1:2,000) (Pierce, USA) for overnight. Finally, membrane was incubated with anti-mouse secondary antibody (1:10,000) for 45 min and was developed using Luminata Forte reagent (Millipore, USA) and immuno reactivity of protein was visualized under chemiluminescence system Image Quant (GE Healthcare Life Sciences, USA).

### Atomic Force Microscopy

UCHL1 was treated with 1, 10 and 50 molar excess of GSNO and diluted with 50 mM Tris-HCl pH 7.4, 50 mM NaCl to a final concentration of 10 μM. For induction of α-synuclein fibrillation, UCHL1 and TM at concentration of 200 μM were treated with 10 molar excess of GSNO and were co-incubated with 800 μM of α-synuclein and α-synuclein mutant A53T at 37 °C and 300 rpm^60^. Samples were aliquoted at 0, 8, 16 and 24 h and were placed on freshly cleaved mica and then air-dried. Samples were imaged in tapping mode by JPK Nano Wizard III atomic force microscope (JPK instrument, Berlin, Germany). The drive frequency of silicon cantilever was between 300–320 kHz and the scan rate was between 0.8–1 Hz with a spring constant of 13–77 N/m. The size of amorphous aggregates was measured from the topographic AFM images with JPK software.

### ANS binding Assay

ANS (8 anilino-1-naphthalene sulfonic acid) (20 μM) was mixed with 10 μM of control and nitrosylated protein in 50 mM Tris-HCl, 150 mM NaCl, pH 7.4 at 37 °C. Increase of ANS fluorescence was monitored at different time intervals using Hitachi F-7000, fluorescence spectrophotometer. The excitation wavelength was 372 nm and the emission wavelength scan was carried out between 400 to 600 nm^61^.

### Thioflavin T (ThT) binding assay

Protein solution was dissolved in 50 mM phosphate, 100 mM NaCl. UCHL1 at concentration of 200 μM was treated with 10 molar excess of GSNO and was co-incubated with 800 μM of α-synyclein at 37 °C and 300 rpm. Readings were taken from these samples after incubating 20 μM of protein with 20 μM of ThT for 5 min. Three independent measurements were performed and subsequently averaged for each sample. ThT fluorescence was recorded using Hitachi F-7000, fluorescence spectrophotometer with excitation at 442 nm and emission scan was recorded from 450 to 600 nm^62^.

### Immunofluorescence microscopy and image analysis

SH-SY5Y cells were treated with rotenone (1 μM) for 16 h and processed for immunostaining. Briefly, cells were fixed in 4% paraformaldehyde solution for 10 min and were blocked with 1% BSA and probed using mouse anti-UCHL1 (1:2,000) antibody (Thermo Fisher Scientific, USA). Secondary antibody, Alexa 488 (Thermo Fisher Scientific, USA) was used at a dilution of 1:1,000. For confocal imaging of mice brain, paraformaldehyde fixed brains of control and rotenone treated mice were processed for cryo-sectioning to obtain 20 μm thick sections. Sections were then processed for immunostaining using reagents from Vector Laboratories, USA. Briefly, after antigen retrieval for 45 min at 70 °C, sections were blocked with 1% BSA and probed using mouse anti-UCHL1 (1:500) and rabbit anti-α-synuclein (1:200) primary antibodies (Santa Cruz, USA). Secondary antibodies, Alexa 590 anti-mouse and Alexa 488 anti-rabbit, were used at a dilution of 1:500. All the fluorescence images were taken as Z-stacks on a confocal microscope (Leica SP5, Germany). Control and experimental samples were imaged with same laser setting and Z-stack thickness. Total numbers of co-localized UCHL1 and α-synuclein positive aggregates were counted using LAS AF Lite software.

### Statistical analysis

Statistical analysis was performed using Graph pad prism software. Values were expressed as standard error of mean (SEM). Data were analyzed using one or two-way ANOVA followed by Bonferroni post hoc test. P-value of < 0.05 was considered statistical significant.

## Additional Information

**How to cite this article:** Kumar, R. *et al*. S-nitrosylation of UCHL1 induces its structural instability and promotes α-synuclein aggregation. *Sci. Rep.* **7**, 44558; doi: 10.1038/srep44558 (2017).

**Publisher's note:** Springer Nature remains neutral with regard to jurisdictional claims in published maps and institutional affiliations.

## Supplementary Material

Supplementary Information

Supplementary Table 1
