# Supplementary material for: S-nitrosylation of UCHL1 induces its structural instability and promotes α-synuclein aggregation
Source: Sci Rep. 2017 Mar 16;7:44558. doi: 10.1038/srep44558 (PMC5353675; doi:10.1038/srep44558)
Supplement: Supplementary Information [file srep44558-s1.doc]

**Manuscript ID: SREP-16-44359-T**

**S-nitrosylation of UCHL1 induces its structural instability and promotes α-synuclein aggregation**

*Roshan Kumar^1, 4^, Deepak K Jangir^1^, Garima Verma^2^, Shashi Shekhar^3^, Pranita Hanpude^1,4^, Sanjay Kumar^1,4^, Raniki Kumari^1^, Nirpendra Singh^5^, Neel Sarovar Bhavesh^2^, Nihar Ranjan Jana^3^ and Tushar Kanti Maiti^1^**

^1^Functional Proteomics Group, Regional Centre for Biotechnology (RCB), NCR Biotech Science Cluster, 3^rd^ Milestone Gurgaon-Faridabad Expressway, Faridabad,121001, India.

^2^Transcription Regulation Group, International Centre for Genetic Engineering and Biotechnology (ICGEB), Aruna Asaf Ali Marg, New Delhi, 110067, India.

^3^Molecular Neuroscience Laboratory, National Brain Research Centre (NBRC), Manesar, Gurgaon, 122051, India.

^4^Manipal University, Manipal, Karnataka, 576104, India.

*^5^*Regional Centre for Biotechnology, NCR Biotech Science Cluster, 3^rd^ Milestone Gurgaon-Faridabad Expressway, Faridabad, 121001, India.

^*^To whom correspondence should be addressed. E-mail: [tkmaiti@rcb.res.in](mailto:tkmaiti@rcb.res.in)

**Supplementary methods**

**Intracellular nitric oxide detection**

DAF-FM diacetate (Invitrogen, USA), a cell-permeable indicator, was used to measure nitric oxide in SH-SY5Y cells treated with rotenone (1µM, 16h). DAF-FM diacetate (10 µM) was added to control and rotenone treated cells for 15 min at room temperature in the dark condition, which were then washed twice with PBS, suspended into PBS and analysed by flow cytometer (Becton Dickinson, USA) using FITC channel.

**Nitrosoproteome analysis in PD mice brain**

Alkylating biotin switch technique was used in mass spectrometry based nitrosoproteome analysis[^1^](#_ENREF_1). Briefly, 2 mg/mL of protein lysate was S-alkylated by adding three volume of blocking buffer (270 mM iodoacetamide in HEN buffer, 2.5% SDS) and incubated at 37 ˚C for 90 min in the dark condition. Excess IAM was removed by acetone precipitation followed by centrifugation at 16,000 ×g for 20 min. The protein pellet was then washed three times with 95% ice-cold acetone and resuspended in HEN buffer with 1% SDS. Protein mixture (2 mg/mL) was simultaneously reduced and labelled in HEN buffer containing 1/50 volume of 250 mM sodium ascorbate and 1/3 volume of 8 mM PEO-iodoacetyl-biotin at 37 ˚C for 2 h in the dark condition. Acetone precipitation was performed to remove excess sodium ascorbate and PEO-iodoacetyl-biotin. Biotinylated protein pellet was resuspended in 50 mM Tris, pH 8.3, 0.1% SDS, 0.02% Triton X-100 and 2 M urea. The protein mixture was reduced with 5 mM TCEP and S-alkylated with a final concentration of 3.5 mM IAM at 37 ˚C for 2 h in dark. The protein mixture was diluted with 50 mM ammonium bicarbonate buffer, pH 8.0 to a final concentration of 1 M urea and digested with trypsin (1:20, trypsin: proteins) at 37 ˚C for 20 h. The peptides were resuspended in (1× PBS, 40 mM NaH_2_PO4 at pH 7.2 and 300 mM NaCl). The resuspended peptides were then mixed with the equilibrated streptavidin agarose beads at a ratio of 1:10 (streptavidin agarose: peptides) and incubated at room temperature for 1 h with gentle agitation. The biotinylated peptides were eluted with the elution buffer contaning 20mM HEPES-NaOH, pH 7.8, 1mM EDTA, 100mM NaCl and 100mM beta mecrcaptop ethanol. The eluted peptides were dried in vaccum, disolved in water with 0.1% formic acid and desalted using Pierce C18 tips (Thermo Fisher Scientific). The peptides were identified in Triplte TOF 5600 system (Sciex, USA) coupled with nano LC. The mass spectrometry data were analyzed in MASCOT(Matrix Science).

**References**

1 Chen, Y. J., Ku, W. C., Lin, P. Y., Chou, H. C. & Khoo, K. H. S-alkylating labeling strategy for site-specific identification of the s-nitrosoproteome. *Journal of proteome research* **9**, 6417-6439, doi:10.1021/pr100680a (2010).

**Supplementary Figure S1.**


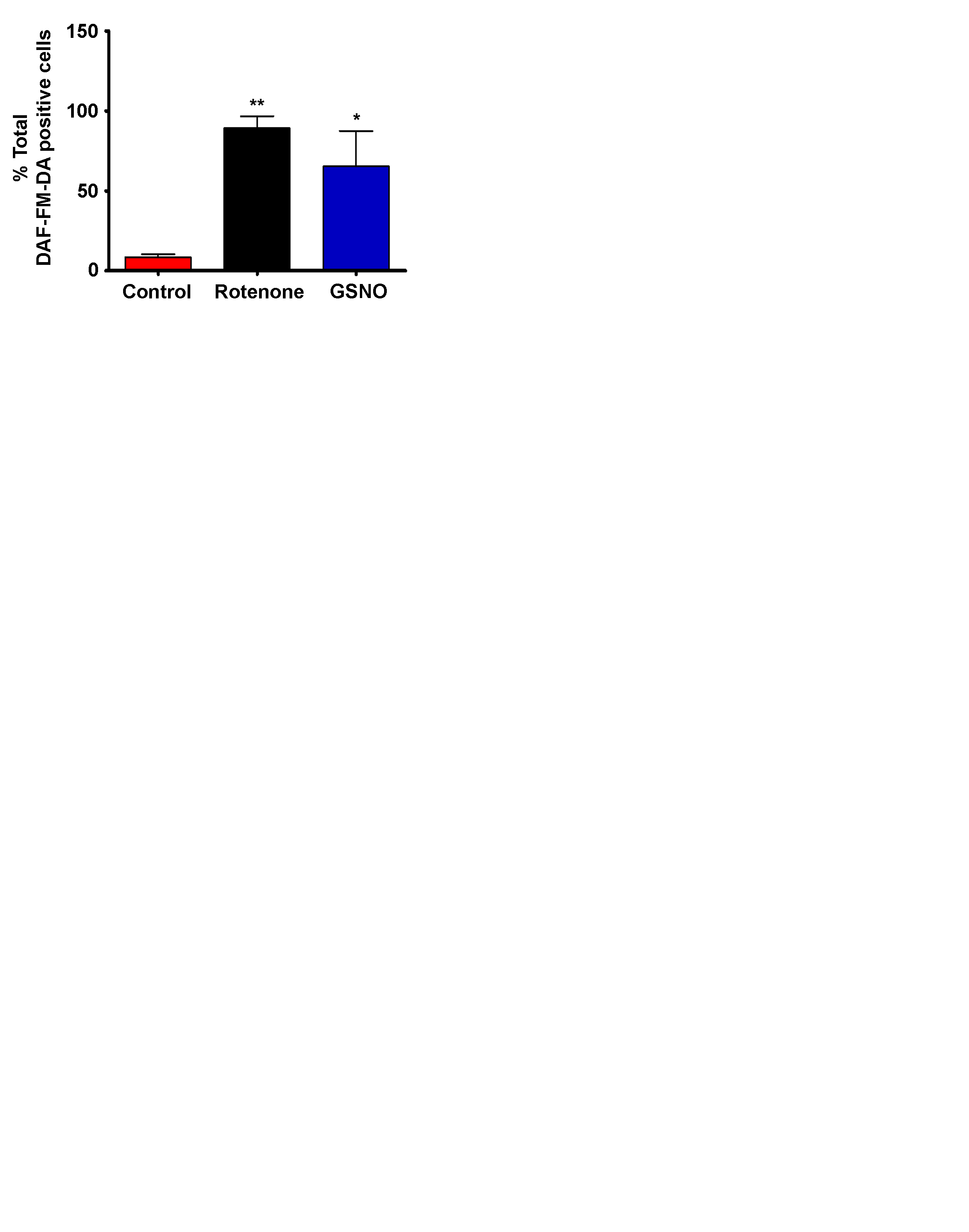


**Supplementary Figure S1. FACS analysis of nitric oxide production.** DAF-FM diacetate, a cell-permeable indicator, was used to monitor nitric oxide in rotenone treated SH-SY5Y cells and in control cells. Rotenone and GSNO treated cells showed approx. 10 and 6 times increase in nitric oxide production respectively.

**Supplementary Figure S2**


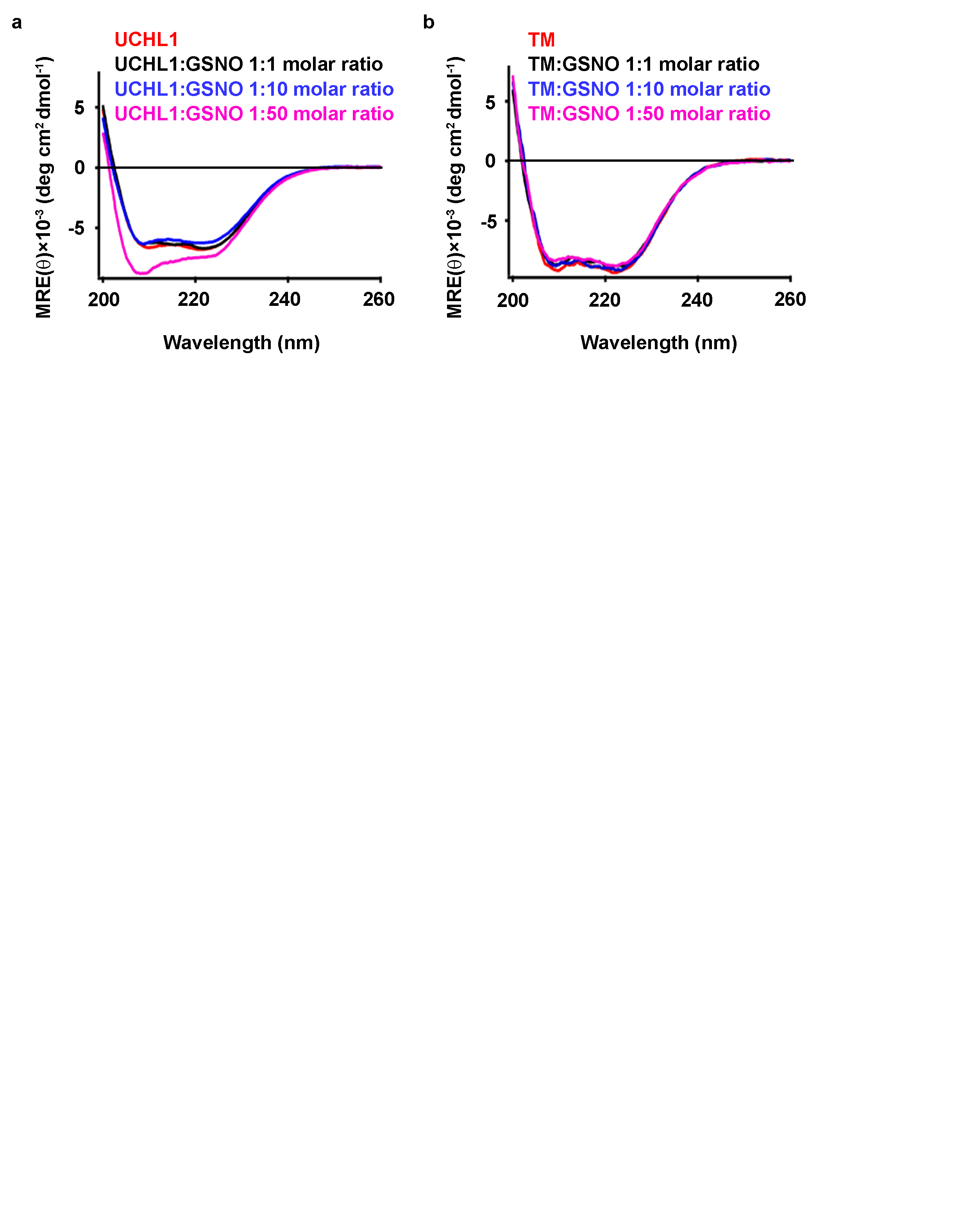


**Supplementary Figure S2. Circular dichroism spectra of nitrosylated UCHL1.** UCHL1 (10 µM) was treated with 1, 10 and 50 molar excess of GSNO at 37 ^0^C for 30 min. CD spectra for (a) control and nitrosylated UCHL1 and (b) TM were recorded from 190 nm to 260 nm using JASCO J815 CD spectrometer

**Supplementary Figure S3.**


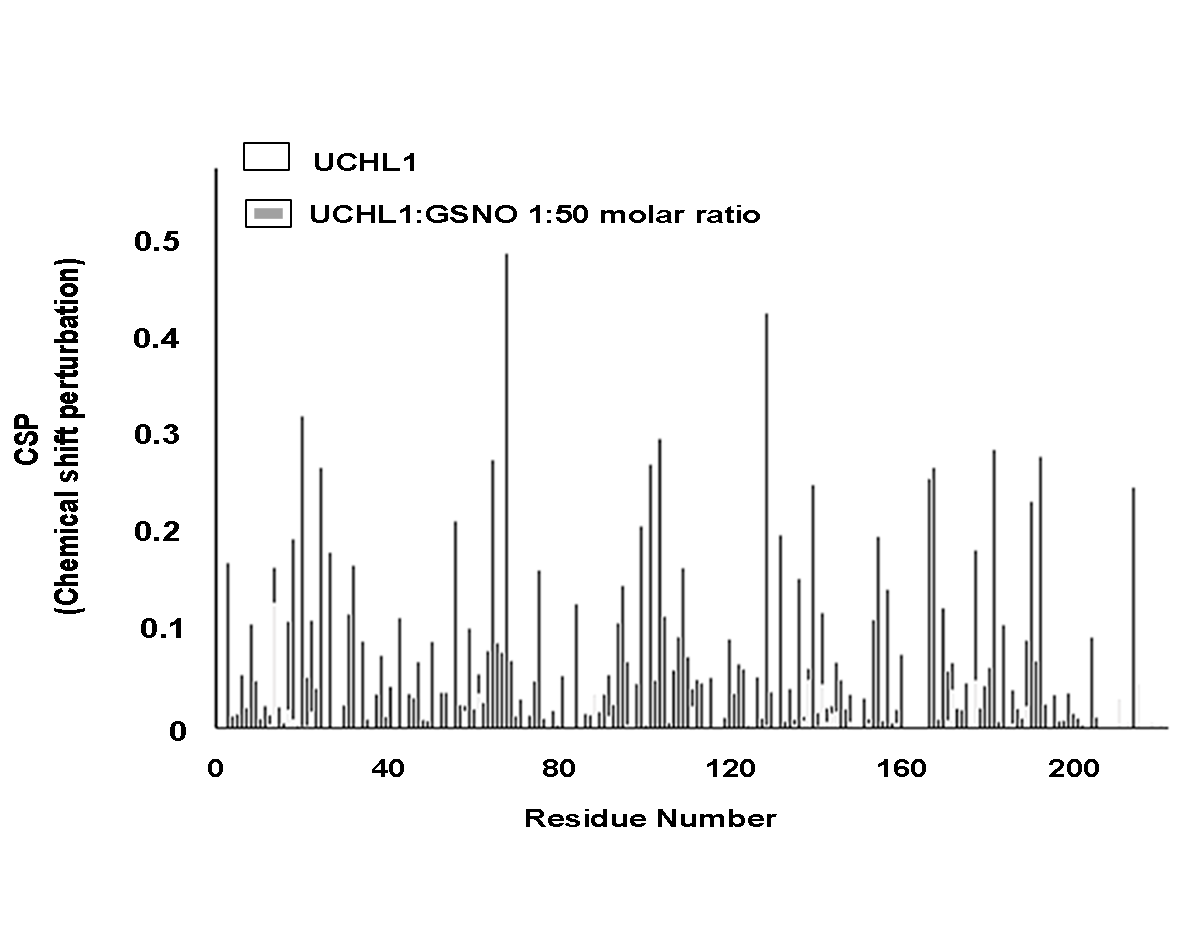


**Supplementary Figure S3.** Chemical shift perturbation (CSP) cumulative backbone amide proton and nitrogen chemical shift perturbation of control and nitrosylated UCHL1

**Supplementary Figure S4.**

**
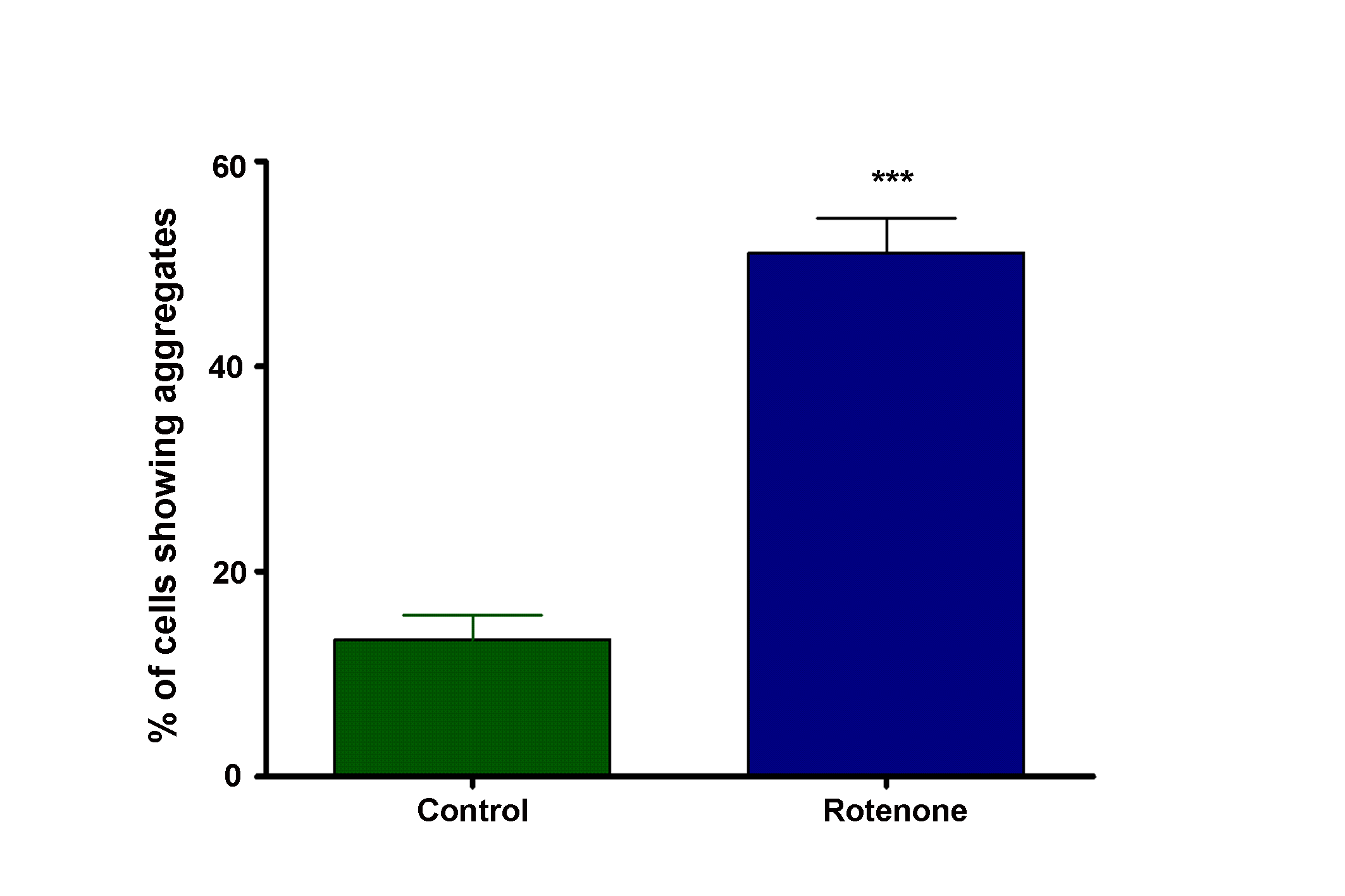
**

**Supplementary Figure S4.** Quantitation of UCHL1 aggregates in SH-SY5Y Cells. Rotenone treated cells showed approx. 48% of cells showing aggregates and this value is statistically significant analyzed by Student T test and ***P value < 0.0001.

**Supplementary Figure S5**


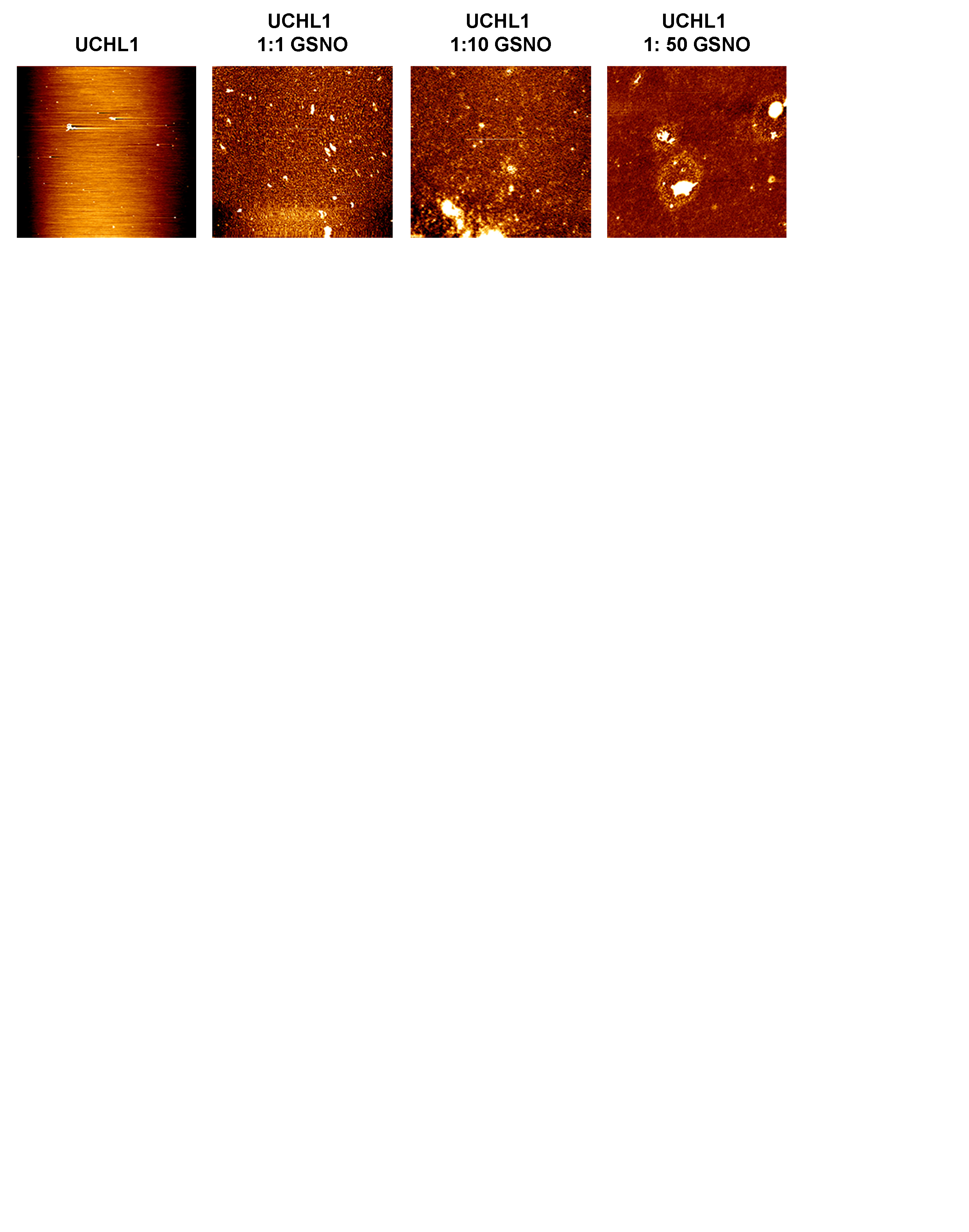


**Supplementary Figure S5. Atomic force microscopy of nitrosylated UCHL1.** UCHL1 (10 µM) was treated with 1, 10 and 50 molar excess of GSNO at 37 ^0^C for 30 min. Proteins were deposited on freshly cleaved mica and air dried. As the concentration of GSNO treatment increased more amorphous aggregates were seen.

**Supplementary Figure S6.**


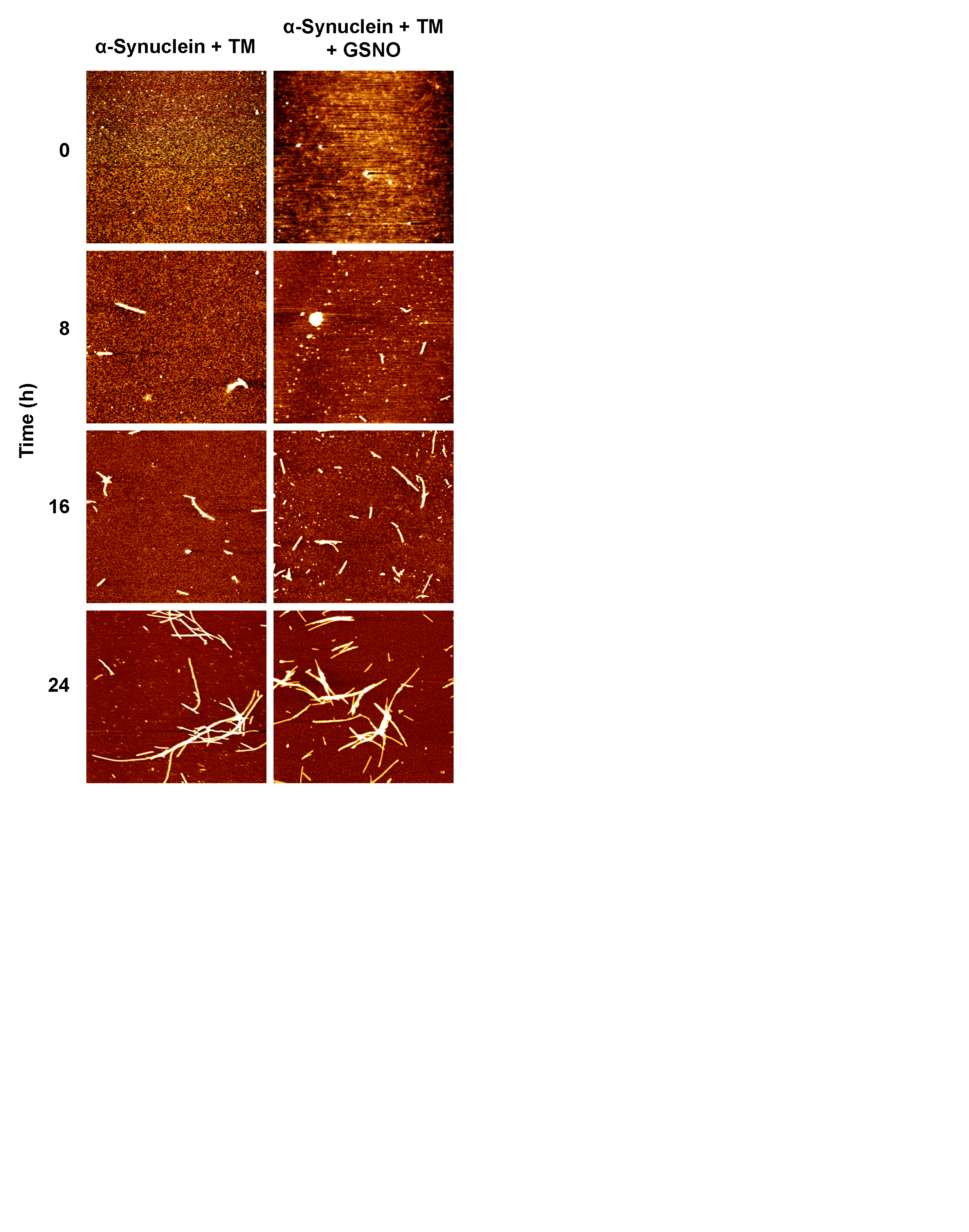


**Supplementary Figure S6. Atomic Force Microscopy.** TM (200 µM) treated with 10 molar excess of GSNO and co-incubated with α-synuclein (800 µM). Samples were aliquoted at 0 h, 8 h, 16 h and 24 h and were placed on freshly cleaved mica and scanned in JPK Nano wizard III software. TM showed similar behavior like wild type and does not induce α-synuclein fibrillation.

**Supplementary Figure S7.**


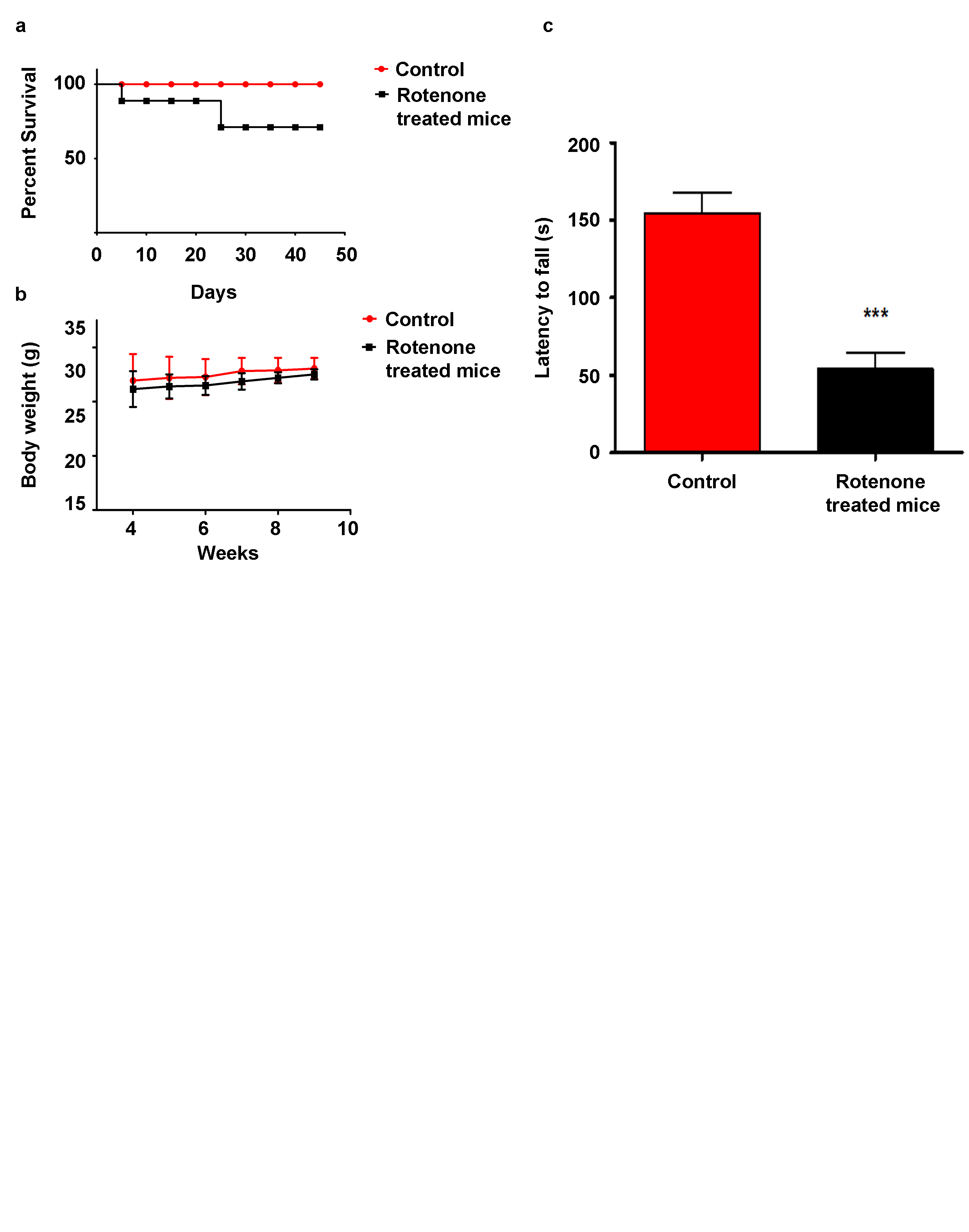


**Supplementary Figure S7. (a) Survival Assay.** Mice were assessed for viability from the age of 6 weeks once daily. Survival data were analyzed by Kaplan–Meier survival analysis. **(b) Body weight.** Control and rotenone treated mice body weight were measured every seventh day. **(c) Rotarod Test.** Mice were placed on the rotarod (Scientific Instruments, New Delhi) and were taught to stay on the rod which was rotating with a constant speed of 5 rpm. Mice that would fall were repeatedly placed back on the rod until they were able to stay on the rotarod for at least 40 sec. Mice were trained for 3 days and then tested at accelerating speed of 2-20 rpm. The performance was recorded when the animal was placed on the rod till when it fell off. Rotenone treated mice fell early compared with control.

**Supplementary Figure S8.**


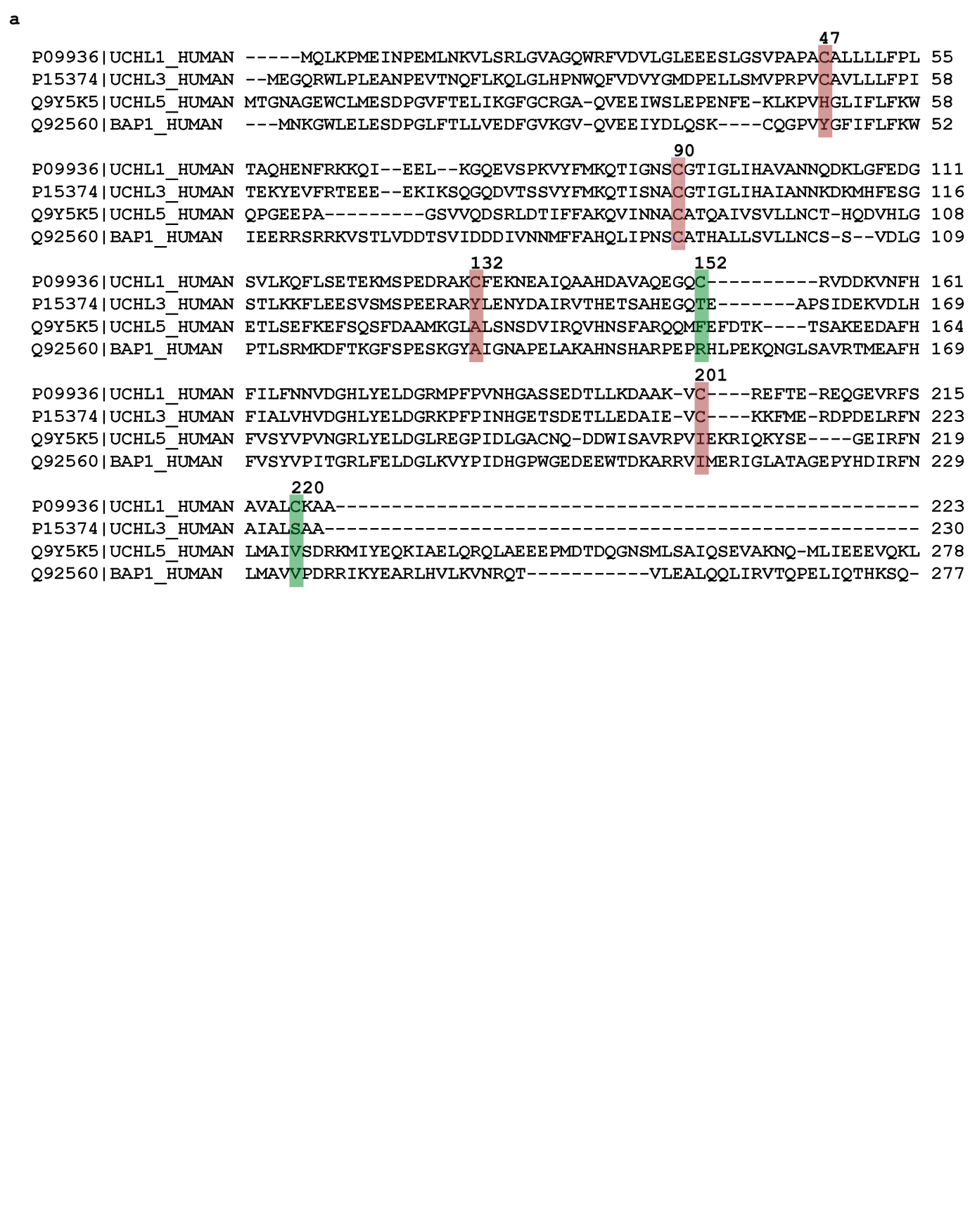


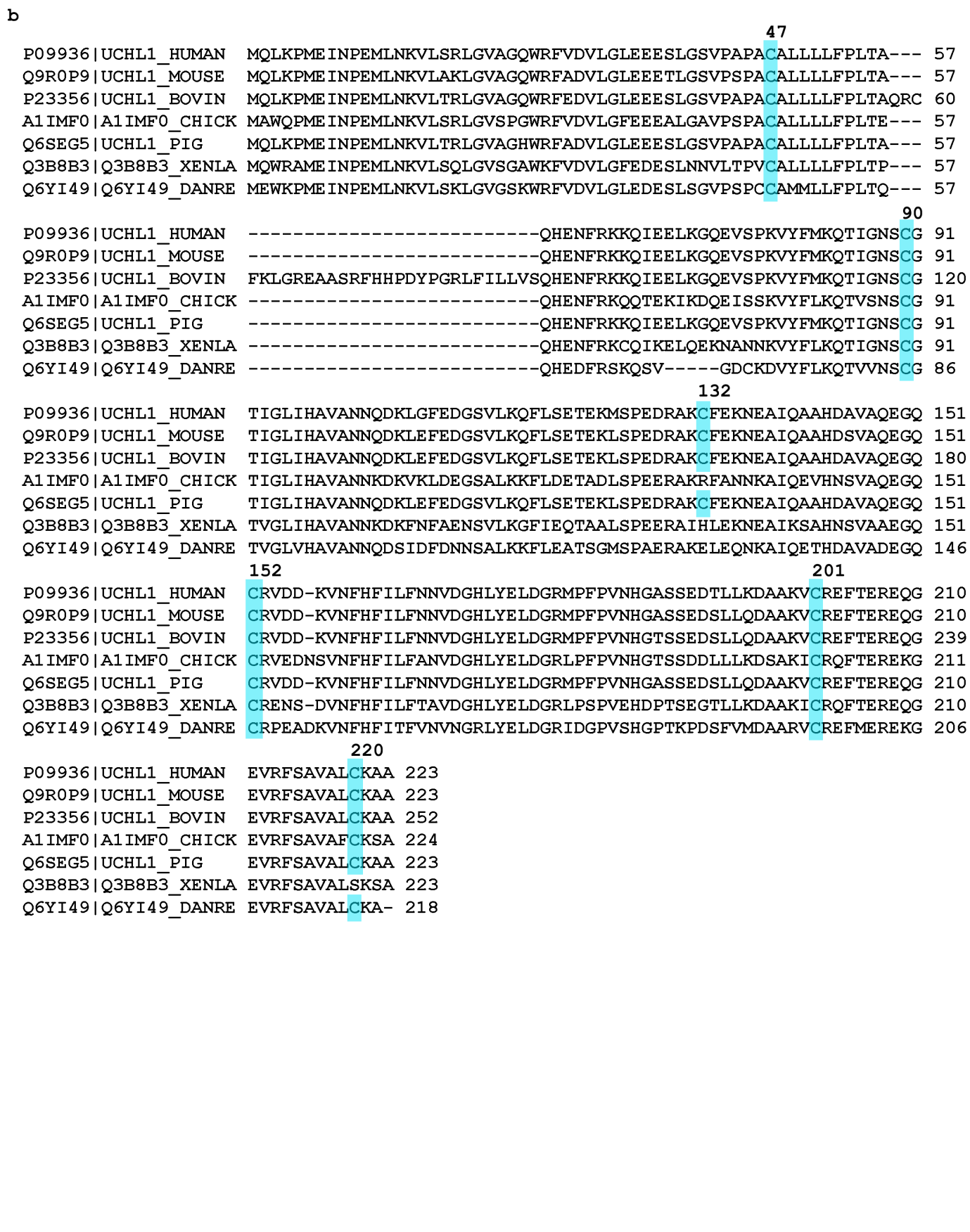


**Supplementary Figure S8**. **Cysteine 152 and 220 are specific to UCHL1. (a)** Sequence alignment of UCHL1 between different species depicts that cysteine of human UCHL1 is highly conserved among species like mouse (MOUSE), bovine (BOVIN), chicken (CHICK), Sus scrofa (PIG), Xenopus (XENLA), zebra fish (DANRE). Conserved cysteine residues are highlighted in cyan blue. **(b)** Sequence alignment of UCHL1 with other UCH family members shows cysteine 152 and 220 (highlighted in green) are specific to only UCHL1 and not found in other family members. Other cysteine residues are highlighted in red.

**Supplementary Figure 9**


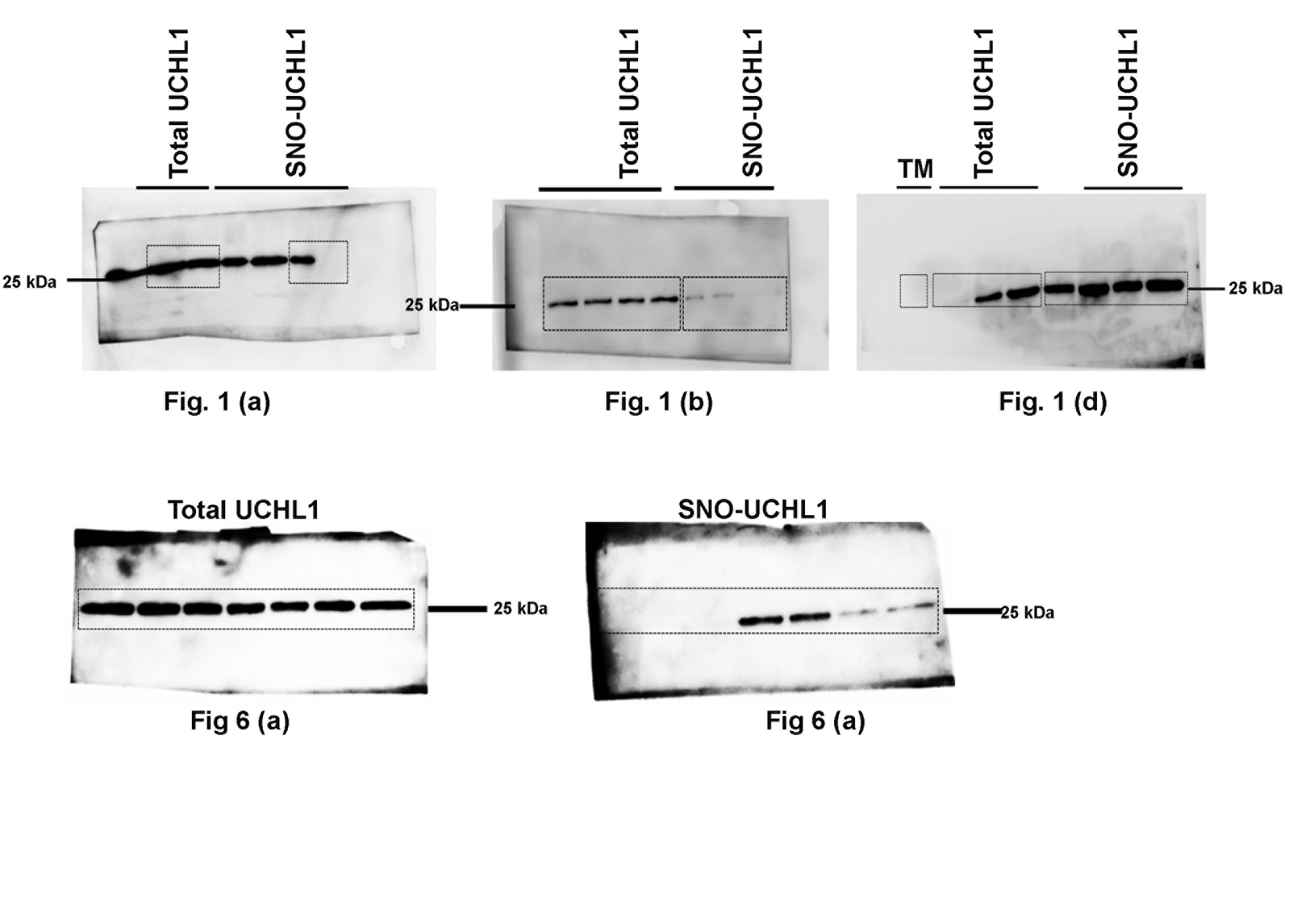


**Supplementary Figure 9.** Uncut WB image of nitrosylated UCHL1 in SH-SY5Ycells, purified protein and mouse brain as presented in the **Figures 1** and **Figure 6.**

**Supplementary Table 1.** Nitrosoproteome analysis of rotenone treated SH-SY5Y cells and rotenone induced PD mice brain (Supplementary excel sheet).

**Supplementary Table S2.**

| **Sample** | **Nitrosylated cysteine position** | **Peptide sequence** | **Modification** |
| --- | --- | --- | --- |
| UCHL1 | - | - | - |
| UCHL1:GSNO 1:1 molar ratio | 152 | NEAIQAAHDAVAQEGQCR | HPDP Biotin |
| UCHL1:GSNO 1:10 molar ratio | 90,152 and 220 | QTIGNSCGTIGLIHAVANNQDK  NEAIQAAHDAVAQEGQCR  FSAVALC | HPDP Biotin |
| UCHL1:GSNO 1:50 molar ratio | 90,152 and 220 | QTIGNSCGTIGLIHAVANNQDK  NEAIQAAHDAVAQEGQCR  FSAVALC | HPDP Biotin |

**Supplementary Table S2. Concentration dependent nitrosylation of recombinant UCHL1.** UCHL1 was treated with 1, 10 and 50 molar excess of GSNO. Biotin switch assay was performed to replace NO moiety with HPDP biotin. MALDI MS spectrometry was performed to identify nitrosylation site. With 10 and 50 molar excess of GSNO, C90, C152 and C220 get modified whereas with 1 molar excess of GSNO only C152 gets modified.
